# Misactivation of multiple starvation responses in yeast by loss of tRNA modifications

**DOI:** 10.1093/nar/gkaa455

**Published:** 2020-06-02

**Authors:** Alexander Bruch, Teresa Laguna, Falk Butter, Raffael Schaffrath, Roland Klassen

**Affiliations:** Institut für Biologie, Fachgebiet Mikrobiologie, Universität Kassel, Heinrich-Plett-Str. 40, 34132 Kassel, Germany; Department of Quantitative Proteomics, IMB Mainz, Ackermannweg 4, 55128 Mainz, Germany; Department of Quantitative Proteomics, IMB Mainz, Ackermannweg 4, 55128 Mainz, Germany; Institut für Biologie, Fachgebiet Mikrobiologie, Universität Kassel, Heinrich-Plett-Str. 40, 34132 Kassel, Germany; Institut für Biologie, Fachgebiet Mikrobiologie, Universität Kassel, Heinrich-Plett-Str. 40, 34132 Kassel, Germany

## Abstract

Previously, combined loss of different anticodon loop modifications was shown to impair the function of distinct tRNAs in *Saccharomyces cerevisiae*. Surprisingly, each scenario resulted in shared cellular phenotypes, the basis of which is unclear. Since loss of tRNA modification may evoke transcriptional responses, we characterized global transcription patterns of modification mutants with defects in either tRNA^Gln^_UUG_ or tRNA^Lys^_UUU_ function. We observe that the mutants share inappropriate induction of multiple starvation responses in exponential growth phase, including derepression of glucose and nitrogen catabolite-repressed genes. In addition, autophagy is prematurely and inadequately activated in the mutants. We further demonstrate that improper induction of individual starvation genes as well as the propensity of the tRNA modification mutants to form protein aggregates are diminished upon overexpression of tRNA^Gln^_UUG_ or tRNA^Lys^_UUU_, the tRNA species that lack the modifications of interest. Hence, our data suggest that global alterations in mRNA translation and proteostasis account for the transcriptional stress signatures that are commonly triggered by loss of anticodon modifications in different tRNAs.

## INTRODUCTION

In all domains of life, tRNAs are subject to extensive post-transcriptional modification ranging from simple methylations to complex nucleobase side chain additions (1). While these chemical modifications can be distributed all over the tRNA, the anticodon stem loop (ASL) represents a modification hotspot (1). ASL nucleosides at position 37 or 34 can be modified by complex chemical side chains which help maintain the open loop configuration by preventing canonical base pairing (position 37) or enhance codon interaction (position 34 and 37). One of these ASL modifications, 5-methoxycarbonylmethyl-2-thiouridine at position 34 (mcm^5^s^2^U34) is added to tRNA^Lys^_UUU_, tRNA^Gln^_UUG_ and tRNA^Glu^_UUC_ by two partially independent pathways in *Saccharomyces cerevisiae*. The Elongator complex (Elp1-Elp6), in concert with Kti11-Kti14 as well as Trm9 and Trm112, introduce the 5-methoxycarbonylmethyl group, while thiolation is accomplished by a second pathway involving Nfs1, Tum1, Urm1, Uba4 and the thiolase complex Ncs2-Ncs6 (2–12). Loss of mcm^5^U34 or s^2^U34 alone or in combination causes various translational defects, including reduced ribosomal A-site binding, enhanced ribosome pausing at critical codons and ribosomal frameshifting (13–18). Suppression of these defects can be achieved by overexpression of tRNA^Lys^_UUU_, tRNA^Gln^_UUG_ and tRNA^Glu^_UUC_, indicating higher-than-normal expression of these hypomodified tRNAs can compensate for malfunctional mRNA translation in response to loss of U34 modifications (19).

Combined absence of mcm^5^U34 and s^2^U34 in *elp6 ncs2* or *elp3 uba4* mutants triggers synthetic growth defects that are thought to result from severe translational defects and associate with enhanced protein aggregation (15,16). Consistent with functional collaboration between tRNA modifications at position 34 and 37, combined modification defects at both sites result in strongly reduced tRNA functionality *in vivo* (16,20,21). For example, lack of mcm^5^/s^2^U34 and cyclic N6-threonylcarbamoyladenosine 37 (ct^6^A37) or pseudouridine 38 (Ψ38) results in severe synthetic growth defects and elevated levels of protein aggregation (16,20). While combined absence of mcm^5^U34 and s^2^U34 impairs tRNA^Lys^_UUU_ and tRNA^Gln^_UUG_, simultaneous loss of mcm^5^/s^2^U34 and either ct^6^A37 or Ψ38 synergistically reduces the function of either individual tRNA alone. This differential effect is due to the fact that only tRNA^Gln^_UUG_ harbors mcm^5^s^2^U34 and Ψ38 modifications, while tRNA^Lys^_UUU_ is uniquely modified with mcm^5^s^2^U34 and ct^6^A37 (22–24). Consistently, synthetic growth phenotypes of mutants deficient in mcm^5^/s^2^U34 and ct^6^A37 (*elp3 tcd1, urm1 tcd1*) can be specifically suppressed by overexpression of tRNA^Lys^_UUU_ whereas strains lacking mcm^5^/s^2^U34 and Ψ38 modifications (*elp3 deg1, urm1 deg1*) are rescued by overexpression of tRNA^Gln^_UUG_ (20). Surprisingly, similar morphological phenotypes, including defects in nuclear segregation and bud-site selection are shared upon elimination of ct^6^A37 (*tcd1*) or Ψ38 (*deg1*) in mutants lacking either mcm^5^U34 (*elp3*) or s^2^U34 (*urm1*) (20).

Apart from direct translational defects, loss of mcm^5^U34 and s^2^U34 has been shown to result in metabolic, proteomic and transcriptomic changes, some of which involve activation of the *GCN4* dependent general amino acid control (GAAC) pathway (13,15,25). Canonical GAAC depends on detection of uncharged tRNA by Gcn2. However, tRNAs lacking mcm^5^/s^2^U34 are expected to be normally charged (26) and apparently activate *GCN4* in a Gcn2 independent manner (13), pointing to a mechanistically distinct mode of GAAC induction in the absence of U34 modifications. Remarkably, not only loss of mcm^5^/s^2^U34 but also t^6^A37 deficiency has been reported to lead to inappropriate GAAC pathway induction without affecting the efficiency of tRNA charging (27–29).

In this study, we took advantage of the differential effects of double mutant combinations on individual tRNAs to characterize shared cellular responses to tRNA malfunction. We demonstrate premature starvation responses are common to mutants with distinct and composite tRNA modification defects. The starvation like state not only involves improper modulation of GAAC gene expression, despite the presence of amino acids, but also includes deregulation of TORC1 controlled transcriptional programs and untimely autophagy onset. Moreover, glucose repression is commonly found to be defective in modification mutants, and the alterations in their transcriptional starvation stress signatures are suppressible by elevating the gene dosage for the tRNA species (i.e. tRNA^Gln^_UUG_ and tRNA^Lys^_UUU_) that are undermodified in the mutant strains of interest. Together with our findings showing that growth phenotypes and enhanced protein aggregation can be suppressed by tRNA overexpression, we consider cell growth, protein homeostasis and timely activation of starvation responses appear all to be linked to proper tRNA anticodon modifications.

## MATERIALS AND METHODS

### Strains, plasmids and general methods

Yeast strains used and generated throughout this study are listed in Supplementary Table S1. Gene deletions were performed by PCR mediated protocols based on the pUG plasmid set (30) with primers listed in Supplementary Table S2. Verification of gene deletions were carried out with forward/reverse primers positioned outside of the target loci (Supplementary Table S2). The different strains were cultivated in synthetic minimal (YNB) or complex rich (YPD) media using standard methods (31). Rapamycin (Sigma, USA) treatment of yeast cultures involved 0.2 μg/ml for 3 h. For overexpression of tRNA^Gln^_UUG_ and tRNA^Lys^_UUU_, we used multi-copy plasmids pRK55 and pDJ83 (20,32). These are based on pRS425 and YEplac181, respectively, which also served as empty vector in control experiments. Expression of the TORC1-dependent GFP-Atg8 reporter fusion was done with plasmid pRS315-*GFP-ATG8* kindly provided by Drs Cha and Corcoles (33). HA tagged Atg13 was expressed from pRS316-*ATG13-2HA* which was made available by Drs Yamamoto and Hatakeyama (34). Yeast strains were generally transformed with plasmids according to the protocol by Chen *et al.* (35).

### RNA sequencing

Yeast cultures were incubated until an OD_600_ = 1.0 was reached, and total RNA was isolated using the RNeasy Mini Kit (Qiagen, Germany) following the instructions of the manufacturer. NGS library prep was performed with Illumina's TruSeq stranded mRNA LT Sample Prep Kit following Illumina's standard protocol (Part # 15031047 Rev. E). Libraries were prepared by using only ¼ of the reagents with a starting amount of 250ng and they were amplified in 11 PCR cycles. Libraries were profiled in a High Sensitivity DNA on a 2100 Bioanalyzer (Agilent technologies) and quantified using the Qubit dsDNA HS Assay Kit, in a Qubit 2.0 Fluorometer (Life technologies). Samples were pooled with other samples in equimolar ratio and sequenced on different HiSeq 2500 lanes, SR for 1 × 58 cycles plus 2 × 8 cycles for the index read, and with a read length of 75 bp in single end mode.

### Analysis of differentially expressed genes

RNAseq data (fastq files) analysis was performed using the RNAseq pipeline developed by the IMB’s (Institute of Molecular Biology, Mainz) Bioinformatics core facility (https://github.com/imbforge/NGSpipe2go). Briefly, mapping was performed using STAR (v.2.5.1b) (36,37) against yeast genome v. R64-1-1.84. Counts per gene were derived using htseq count (v.1.4.6-p2) (38). Differential expression analysis between each mutant type and wt species was done using DESeq2 (v.1.10.1) (39), which works by using generalized linear models based on negative binomial distributions. Differential expressed genes were filtered for an FDR of 1%. Venn diagrams depicting overlap between differentially expressed genes in all the 4 mutants were generated using the R package VennDiagram (40).

### Gene ontology analysis

Analysis to investigate the significantly affected biological routes was carried out using Gene Ontology website (http://geneontology.org/) (41), obtaining significant pathways in the three categories *Biological Proccess*, *Molecular Function* and *Cellular Component*. Later, results pathways were grouped according to their GO families using REVIGO algorithm (42). Plots were produced using R package ggplot2 (43).

### Isolation of total RNA and mRNA quantification by qRT-PCR

Yeast strains were cultured until an OD_600_ = 1.0 was reached, and then subjected to total RNA isolation. The cell pellet was resuspended with 500 μl of NucleoZOL (Macherey-Nagel, Germany) and 100 μl of acid-washed glass beads. Samples were strongly vortexed for 1 min and cooled for 5 min. This procedure was repeated five times. Next, the suspensions were mixed with 100 μl chloroform and incubated for 5 min at room temperature. Following centrifugation for 5 min at 15 000 rpm, the cell lysate was separated into three phases and the clear one was transferred into a new tube and mixed with 200 μl chloroform. Repeating the incubation and centrifugation step led to a clearer phase, which was mixed in a new tube with 250 μl precooled 100% isopropanol. The samples were incubated on ice for 15 min and centrifuged for 5 min at 15000 rpm to pellet the RNA. The pellets were washed with 70% ethanol and solved in 50 μl DEPC–H_2_O. Remnant DNA in the isolates was degraded with RNase-free DNase I (ThermoFischer, USA) and checked via PCR for possible DNA contamination. Finally, the prepared RNA was used in qRT-PCR reaction mixtures. Quantification of *HXK1*, *MEP2*, *HSP12, SRP21* and *ACT1* mRNA levels from different strain backgrounds (Supplementary Table S1) and grown under varying conditions was done with a Mastercycler ep realplex (Eppendorf, Germany) using the Luna^®^ Universal One-Step RT-qPCR Kit (NEB, USA) and gene specific oligonucleotides (Supplementary Table S2) utilizing three biological replicates and applying technical triplicates for each sample, respectively. The transcript levels for each gene were normalized to *ACT1* mRNA quantities measured as previously described (44). Statistical significance was determined using two-tailed *t*-test.

### Protein isolation and Western blots

Yeast cultures harvested at OD_600_ = 1.0 were subjected to total protein extraction using glass beads as described (45). Protein concentration of cleared cell lysates was measured with the Bradford assay and equal amounts of total protein extracts from different strains were used for standard SDS-PAGE. Western blots utilized anti-GFP (Santa Cruz Biotechnology, USA), anti-HA (ThermoFisher, USA) and anti-Cdc19 antibodies (kindly provided by Dr J. Thorner).

### Isolation of protein aggregates

Yeast strains were cultivated until OD_600_ = 1.0 and 50 OD_600_ units harvested. Cell pellets were used for isolation of protein aggregates as previously described (46). Protein concentrations of lysed and cleared extracts were quantified with the Bradford assay, and all samples were adjusted to the same amount of proteins before continuation of isolation (46). The aggregate pellets were solved in 20 μl H_2_O, mixed with 5× SDS sample buffer and boiled for 5 min. 15 μl of each sample were loaded onto a NuPAGE Bis–Tris gradient (4–12%) gel (ThermoFisher, USA) and separated for 2 h at 100 V. In addition, control experiments involved equal amounts of total protein extracts that were separated on NuPAGE gels under the same conditions.

## RESULTS

### Transcriptome reprogramming in response to loss of different tRNA modifications

Previous work established that composite tRNA modification mutants (*urm1 tcd1*, *elp3 tcd1*, *urm1 deg1* and *elp3 deg1*) display common growth phenotypes and morphological aberrations, which can be attributed to malfunction of distinct tRNA species, i.e. tRNA^Lys^_UUU_ and tRNA^Gln^_UUG_ (2,16,20,21,47). We have demonstrated a translational defect of mRNA encoding a glutamine rich protein in *urm1 deg1* and *elp3 deg1* double mutants (20). To test whether mRNA encoding a lysine rich protein exhibits similar decoding problems in *urm1 tcd1* and *elp3 tcd1* double mutants, we selected *SRP21* (signal recognition particle, 19.16% Lys) for further analysis. Expression was analysed after genomic HA-tagging in the different mutant backgrounds by Western blot. As shown in Supplementary Figure S1A, both *urm1 tcd1* and *elp3 tcd1* double mutants display diminished HA signals whereas Cdc19 (pyruvate kinase) detected as the loading control remained unchanged. We further demonstrated that the expression defect of Srp21-HA can be rescued by overexpression of tRNA^Lys^_UUU_ and that no significant changes in mRNA abundance for *SRP21* were detectable (Supplementary Figure S1B). These results validate our genetic analysis and demonstrate that *urm1 tcd1* and *elp3 tcd1* double mutants carry functionally impaired tRNA^Lys^_UUU_.

Since common phenotypes may be caused by similar transcriptome alterations, we conducted whole transcriptome analysis for the above double mutants to identify a potentially shared response to the loss of the different tRNA modifications.

**Figure 1.**
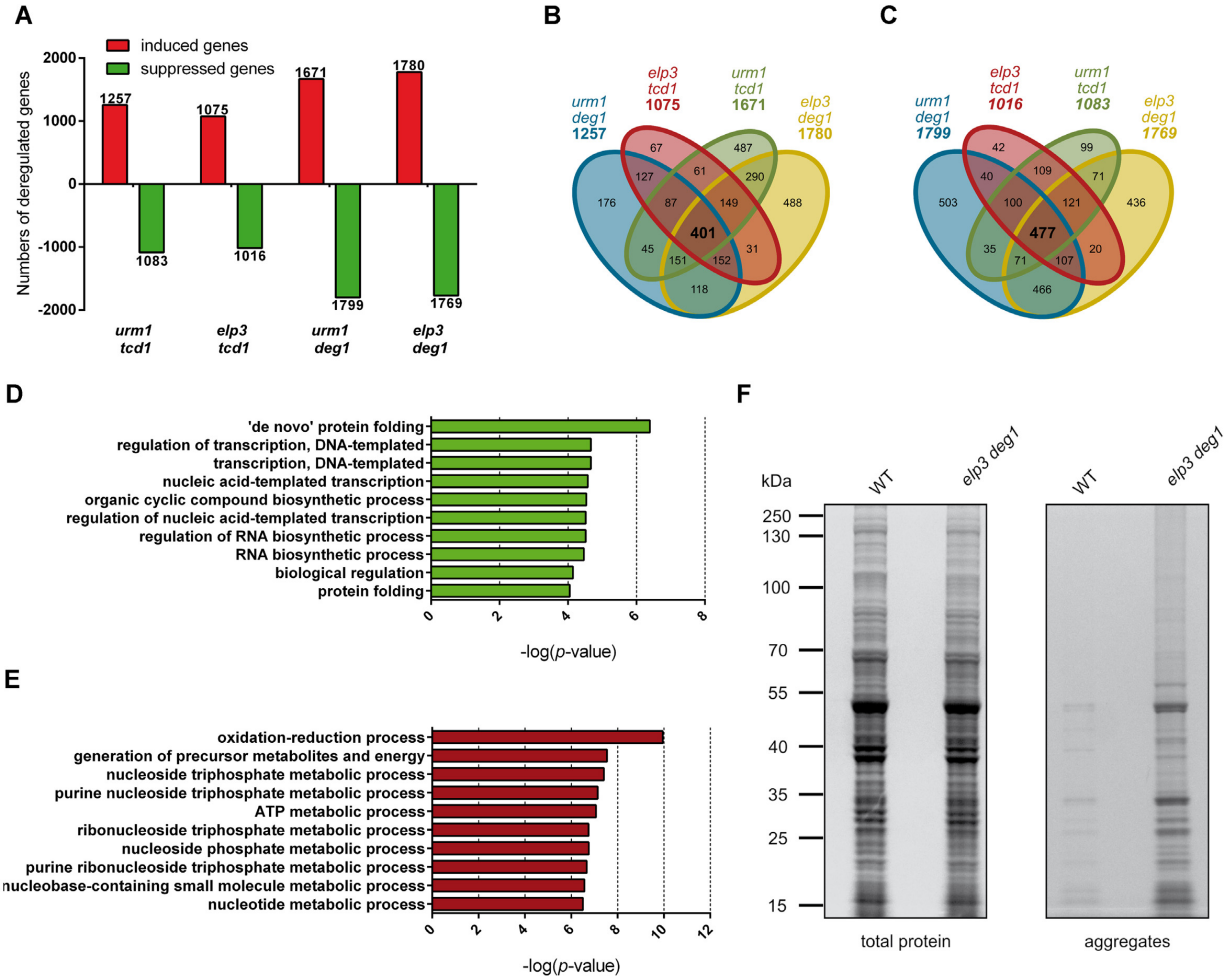
Transcriptome analysis of combined tRNA modification mutants. (**A**) Significantly (adj. *P* ≤ 0.01) up- or downregulated genes identified by RNA sequencing (mutant/WT) are shown for the indicated mutants cultivated in minimal media (YNB). Induced genes are represented in red, suppressed ones in green. Venn diagrams display overlapping genes (indicated in bold black) that were induced (**B**) and suppressed (**C**) in the set of indicated mutants. Commonly deregulated genes were functionally assigned to Gene Ontology (GO) terms (94,95). Listed are ten GO terms with the highest significance of commonly suppressed (**D**) and induced (**E**) genes. (**F**) Wild type (WT) and *elp3 deg1* mutant cells cultivated in minimal (YNB) media were subjected to isolation of protein aggregates as described previously (46). The same amount of protein extract for each strain (left panel) was used for detection of protein aggregates (right panel) by SDS-PAGE and Coomassie staining.

We grew our set of tRNA modification mutants to early exponential phase for subsequent RNA isolation and sequencing analysis. In the different mutants, 1075 to 1780 genes were induced and 1016–1769 genes repressed at the mRNA level (Figure 1A, Supplementary Table S3). Remarkably, in the background of our double mutant set, 401 genes were commonly induced (Figure 1B, Supplementary Table S4) and 477 genes repressed in all four mutants (Figure 1C, Supplementary Table S5).

Upon further examination by gene ontology analysis, many of the upregulated genes were associated with GO terms related to ATP metabolism and energy generation by respiration (Figure 1E; Supplementary Figure S2, Table S6). Several of the induced genes are normally repressed in exponential phase and only expressed after the diauxic shift (e.g. *TSL1*, *GSY2*, *GLC3*) (Supplementary Table S12) or under conditions of activated respiration (*COX3*, *COX4*, *COX5A*, *COX5B*, *COX13*, *COX20*, *ATP3*, *ATP4*, *ATP7*, *ATP16*, *ATP17)* (Supplementary Tables S6 and S13). The induced gene groups are involved in storage carbohydrate synthesis or ATP generation and their induction suggests a mis-sensing of the nutrients present in the growth media. In addition, the upregulated genes are highly enriched in the GO cellular components related to mitochondria (‘mitochondrion’, ‘mitochondrial part’, ‘mitochondrial envelope’, ‘mitochondrial respiratory chain’, ‘mitochondrial membrane’, etc., all significant with *P*-values < 1 × 10^−7^) (Supplementary Table S10). Interestingly, mutants defective in mcm^5^/s^2^U modification were already identified to exhibit mitochondrial translation defects at elevated temperature, which result in respiration deficiency (48). Also, *tcd1* mutants were found to be defective in respiratory growth (24). We confirmed complete absence of respiratory growth for the combined *elp3 tcd1* double mutant and further observe that overexpression of tRNA^Lys^_UUU_, which restores expression of lysine rich Srp21 and suppresses the temperature sensitive growth phenotype, does not enable respiratory growth in the mutant (Supplementary Figure S3). Hence, the transcriptional activation of mitochondrial genes involved in respiration and ATP generation occurs in different combined modification mutants along with a negative effect on respiratory growth.

In addition to respiration genes, mitophagy and autophagy (*P*-values = 8.5 × 10^−3^ and = 7.2 × 10^−3^, respectively) related genes are commonly upregulated (Supplementary Figure S2) in the combined tRNA modification mutants and several TOR controlled genes such as *DAL3, GAT1, GDH1, GLN1, MEP2, MEP3* (Supplementary Table S14) are strongly induced in each of the mutants, pointing to a derepression of nitrogen catabolite repression (NCR) genes during exponential phase in the presence of ammonium. Since a derepression of GAAC controlled genes has been observed in other tRNA modification mutants (13,15,27,49), we checked for deregulation of amino acid biosynthesis genes. Interestingly, in different amino acid biosynthesis pathways, individual *GCN4* controlled genes are upregulated while other genes of the same pathway are downregulated (Supplementary Figure S4). This finding points to a subtle disturbance of GAAC which is distinct from canonical *GCN4* induction upon amino acid starvation.

By evaluating the biological processes significantly enriched in the down-regulated genes in the four mutants, it turned out that 233 (of 477) genes are involved in transcriptional control (e.g. *POL5*, *MOT2*, *RPC53*, *SNF5*, *RPC82*, *RBA50*), translational machinery function (e.g. *ANB1*, *RPG1*, *RBG2*, *ZUO1*, *DYS1*) and respiratory/stationary growth phase (e.g. *HDA2*, *HXK2*, *STT4*, *CAB2*) (Figure 1D, Supplementary Figure S2, Table S8). These gene groups are normally repressed after the diauxic shift (i.e. upon glucose depletion). In contrast to the wild type pattern, their transcription in the tRNA modification mutants is already decreased in exponential growth phase, again resembling transcriptional changes in response to acute starvation (50,51). Such transcriptional signatures are remarkable since *S. cerevisiae* switches only after glucose depletion to respiration followed by stationary phase entry (50,51). A closer inspection of suppressed genes sorted to the significantly enriched (*P*-value = 3.42 × 10 ^−5^, Supplementary Table S7) GO term ‘RNA biosynthesis’ revealed the presence of transcription factors which could mediate part of the observed transcriptional changes (Supplementary Table S11). For example, *HXK2*, *CYC8* and *NRG1* encode proteins directly involved in glucose repression (52–54). Also, several downregulated genes in this category are relevant for chromatin remodelling, histone deacetylation or encode components of the mediator complex, suggesting that the RNA polymerase lI transcription machinery is negatively affected. Taken together, our data indicate that regardless of nutrient conditions, the different tRNA modification mutants typically share with each other a premature and inappropriate induction of genes that are normally activated during starvation and stationary phase.

Surprisingly, downregulated genes are also found to be enriched in GO categories relevant to protein folding, which is counterintuitive due to the previously observed tendency of the mutants to accumulate protein aggregates (20). We confirmed protein aggregation in the analysed mutants (see below) under the applied growth conditions for transcriptome profiling (Figure 1F). Hence, downregulation of protein folding genes may well contribute to the observed protein aggregation. Other than the majority of protein folding relevant genes, *HSP26* encoding a chaperone suppressing protein aggregation (55) is commonly and strongly induced in all four mutants analysed in this study (Supplementary Tables S3 and S15). In addition, *HSP12* encoding a membrane associated heat shock protein (56) is the strongest upregulated gene for three of these (Supplementary Tables S3 and S15). Thus, resembling the transcriptional deregulation of amino acid biosynthesis pathways, protein folding relevant and heat shock induced genes are similarly altered with a strong induction of individual members of this class (*HSP12* and *HSP26*) and concomitant downregulation of others (including *HSP104, HSP82, HSP42, HSP60, SSA1, SSA2*, Supplementary Table S15). Since *HSP12* and *HSP26* are not only induced by heat shock but also upon other stresses (57,58) including glucose starvation, their strong induction in the different tRNA modification mutants may again result from untimely starvation response activation in exponential growth phase.

### Premature stationary phase marker induction by loss of tRNA modifications

**Figure 2.**
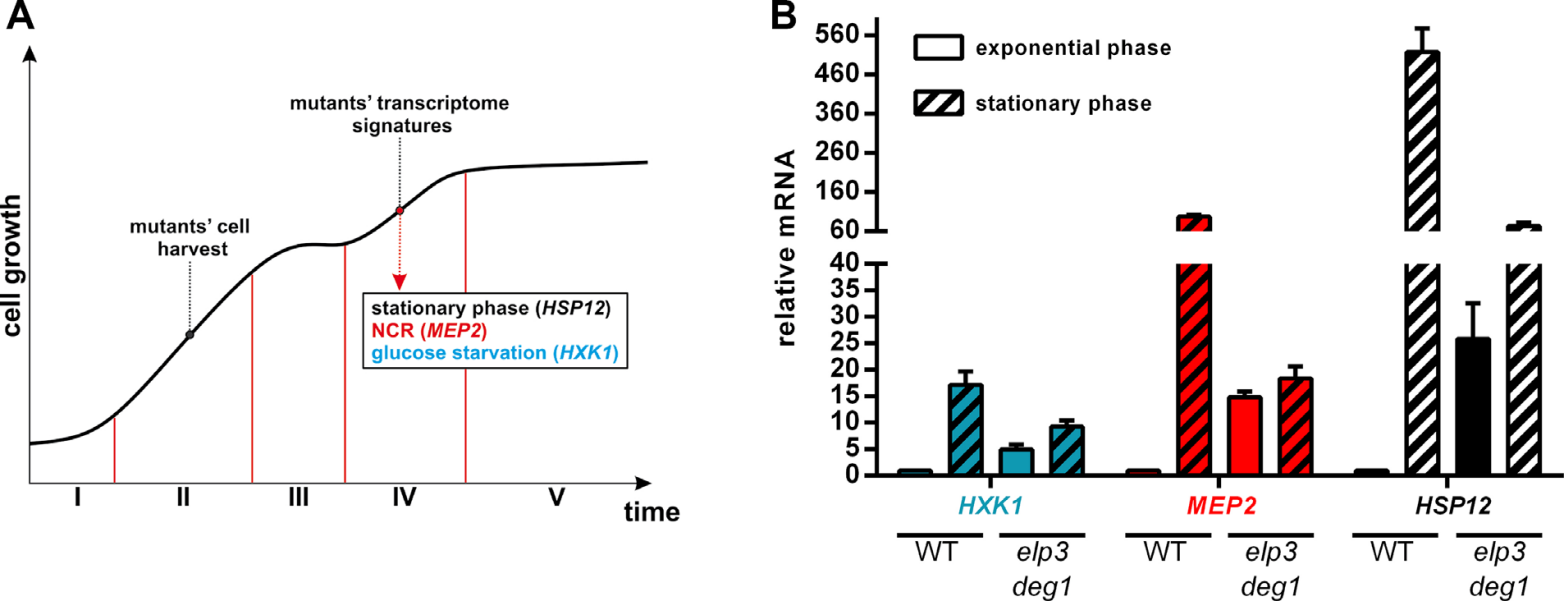
Combined tRNA modification mutants display inappropriate activation of the stationary phase programme during exponential growth. (**A**) Schematic growth curve of *S. cerevisiae* subdivided in five common growth phases: lag-phase (I), exponential-phase (II), diauxic shift (III), post diauxie (IV) and stationary phase (V) (51). Transcriptome profiling of tRNA modification mutants collected in early exponential growth phase (black dot) revealed signatures of the post-diauxic/stationary growth phase (red dot, arrow and box). (**B**) To examine growth specific transcriptional differences due to tRNA modification loss, the indicated mutants were cultivated until exponential (OD_600_ = 1.0) and stationary growth phases. Total RNA was isolated from biological triplicates and each subjected to qRT-PCR as three technical replicates using marker genes representative for NCR (*MEP2*), glucose starvation (*HXK1*) and general stress (*HSP12*). The resulting relative mRNA levels are represented by blank bars for exponential growth phase and checked bars for stationary phase, respectively.

During exponential growth, yeast relies on fermentation for energy generation and upon the diauxic shift, switches to a respiratory lifestyle. Decreasing nutrient availability forces the cell to activate starvation/stationary phase transcriptional programs and autophagy (Figure 2A) (51,59). This adaptation is necessary to reduce translation and induce expression of proteins required to assess alternative nutrient sources and cope with starvation stress (51,60,61).

Since tRNA modification mutants display in exponential phase transcriptome signatures diagnostic for stationary phase (Figure 1D and E, Supplementary Tables S8 and S9), we compared the induction of specific starvation responsive marker genes between the different growth stages (Figure 2A). We quantified mRNA levels for genes diagnostic for the general stationary phase response (*HSP12*) (58), NCR (*MEP2*) (62) and glucose starvation (*HXK1*) (63,64) in early exponential and stationary growth phases. In exponential phase, the tRNA modification mutant *elp3 deg1* induced the expression of *HXK1* ∼ 5-fold, *MEP2* 15-fold and *HSP12* 30-fold (Figure 2B blank bars). These values largely confirm the findings of our transcriptome analysis (Figure 1, Supplementary Table S3). In stationary phase, the WT displayed a strong induction of all three marker genes (*HXK1*: 15-fold, *MEP2*: 95-fold, *HSP12*: 500-fold) (Figure 2B checked bars) when compared to transcription levels in exponential phase. This pattern neatly reflects reports on derepression of the different starvation pathways upon nutrient limitation (51,59). In stationary phase, the *elp3 deg1* mutant also displayed higher relative mRNA levels for *HXK1*, *MEP2* and *HSP12* (checked bars) but when compared to exponential phase, only a moderate further increase (1.5- to 3-fold) was detectable (Figure 2B, Supplementary Table S3).

Taken together, mRNA quantification of the starvation marker genes supports our transcriptome analysis results, demonstrating that mutants with combined tRNA modification defects almost fully activate starvation and stress response pathways during the exponential growth phase. Moreover, and consistent with a defect in transcriptional programming, further gene induction is only weakly increased when the tRNA modification mutants have actually entered stationary phase.

### Defective TOR signalling and autophagy misactivation in response to tRNA modification loss

**Figure 3.**
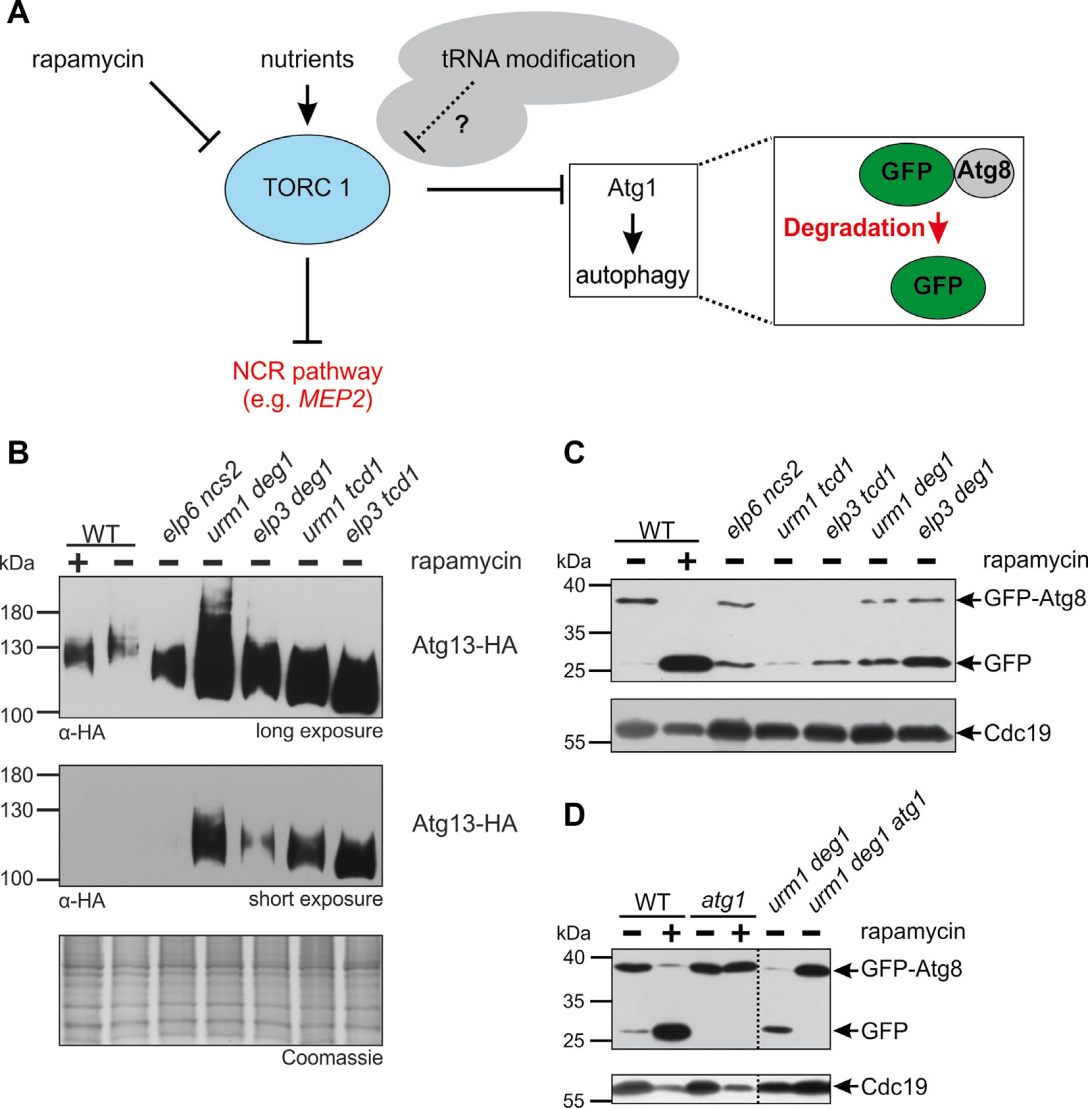
Premature induction of autophagy in tRNA modification mutants. (**A**) Scheme depicting TORC1 regulation by nutrient availability or rapamycin and effects on NCR and autophagy. Arrows indicate activation, blunt-end lines inhibition. tRNA modification may additionally influence TORC1 activity (stippled line). Processing of a GFP-Atg8 fusion protein is commonly used to monitor autophagy. (**B**) The indicated strains were transformed with a plasmid carrying the gene coding for Atg13-HA and were cultivated in minimal media until OD_600_ = 1.0. In contrast to the other strains (−), the wild type (WT) was additionally treated with rapamycin (0.2 μg/ml for 3 h) (+). Detection and discrimination of the phosphorylation status of Atg13-HA was achieved utilizing anti-HA antibodies and equal loading was confirmed via Coomassie staining of the gel. Two different exposure times (long and short) were used due to the variation in signal intensity in the different samples. (**C**) Immunodetection of GFP-Atg8 and its proteolysis product from WT and indicated mutants in exponential phase. Inactivation of TORC1 by rapamycin (+) and autophagy induction leads to proteolysis of the GFP-Atg8 fusion and free GFP signals. Equal loading was verified by detection of Cdc19. (**D**) Effect of *atg1* mutation on autophagy induction. WT and indicated mutants were treated with (+) or without (−) rapamycin and autophagy monitored using the GFP-Atg8 fusion protein as above.

One major pathway coordinating cell growth and transcriptome responses with nutrient availability is controlled by the target of rapamycin complex 1 (TORC1) (51,60). In the absence of preferred nitrogen sources, TORC1 becomes inactivated, leading to derepression of NCR genes such as *MEP2* (Figure 3A).

In addition to nitrogen responsive genes, autophagy is also negatively regulated by TORC1 (65). Since our data show that several autophagy related genes are induced upon loss of tRNA modifications in exponential phase (Figure 2B, Supplementary Figure S2B), the modification defects might partially inactivate TORC1 while activating autophagy (66). A direct downstream target of TORC1 which is phosphorylated at multiple positions and related to autophagy is Atg13 (67). We investigated Atg13 phosphorylation in the different tRNA modification mutants in comparison to the untreated and rapamycin treated wild type by electrophoretic mobility shift assays as described earlier (67,68). As shown in Figure 3B, there is indeed a significant downshift of the Atg13 signal after rapamycin treatment and in the untreated tRNA modification mutants, which is consistent with an accumulation of dephosphorylated Atg13 due to TORC1 inactivation. In the tRNA modification mutants, we additionally observe a strong induction in Atg13 signal intensity, which is consistent with the common transcriptional induction of multiple autophagy genes in these mutants (Supplementary Table S3, Figure S2).

To further verify the assumption of activated autophagy, we examined autophagy by using a GFP-Atg8 reporter construct (Figure 3A). Upon autophagy induction, the Atg8 moiety of the GFP fusion protein is degraded and free GFP accumulates, which is detected by Western blot (69,70). We analysed GFP-Atg8 processing in WT and our set of tRNA modification mutants in exponential growth phase. In addition, we included a mcm^5^s^2^U34 defective *elp6 ncs2* mutant (15). As a control, a portion of the WT culture was treated with rapamycin to inactivate TORC1 and consequently induce autophagy. As expected, the WT displayed no degradation of the GFP-Atg8 protein in exponential phase but showed a strong free GFP signal upon chemical inhibition of TORC1 by rapamycin (Figure 3C, lane 1 and 2). Intriguingly, all tRNA modification mutants accumulated detectable free GFP signals during exponential phase, indicative for inappropriate autophagy induction. Notably, rapamycin treatment of the double mutants further increased autophagy dependent GFP-Atg8 degradation (Supplementary Figure S5). However, while the *elp6 ncs2* mutant displayed equal amounts of full length and free GFP signals (Figure 3C, lane 3), *urm1 deg1* and *elp3 deg1* showed stronger GFP accumulation (Figure 3C, lane 6 and 7). The *urm1 tcd1* and *elp3 tcd1* mutants however, also induced degradation of GFP-Atg8 but no full-length protein and strongly reduced GFP signals were detected, possibly due to expression problems. Since the latter two mutants contain functionally impaired tRNA^Lys^_UUU_ and Atg8 is lysine rich (11.1% Lys codons), we assume improper translation of the *GFP-ATG8* mRNA may have compromised normal expression levels of GFP-Atg8. Compromised translation of the mRNA for lysine rich Srp21 was already demonstrated in *tcd1 urm1* and *tcd1 elp3* mutants (Supplementary Figure S1) and a similar effect may apply to *GFP-ATG8* as well.

To test whether inappropriate autophagy in the set of tRNA modification mutants relies on canonical TORC1 signalling, we analysed their dependency on *ATG1* gene function (Figure 3A). *ATG1* encodes a kinase that is essential for autophagy initiation (71). We transformed *atg1* cells and an *urm1 deg1 atg1* triple mutant with the GFP-Atg8 construct and investigated autophagy induction as described above. The *atg1* mutant entirely lost the rapamycin induced free GFP signal, which reflects the necessity of the kinase for the autophagy process (Figure 3D, lane 3 and 4). Interestingly, the free GFP signal indicative for autophagy induction in the *urm1 deg1* mutant (Figure 3C, lane 6) was similarly lost upon *ATG1* deletion (Figure 3D, lane 5 and 6). Thus, improper autophagy induction by loss of tRNA modifications depends on the Atg1 kinase, which itself is under TORC1 control. Collectively, our data thus reveal that in addition to NCR derepression (Figure 2), combined loss of different tRNA anticodon modifications also interferes with other TORC1 controlled starvation responses including autophagy. Consistent with our data are reports on reciprocal regulation of TORC signalling and U34 modifications by Elongator in baker's yeast (66) and fission yeast (72).

### tRNA overdoses suppress improper starvation responses and protein aggregation

**Figure 4.**
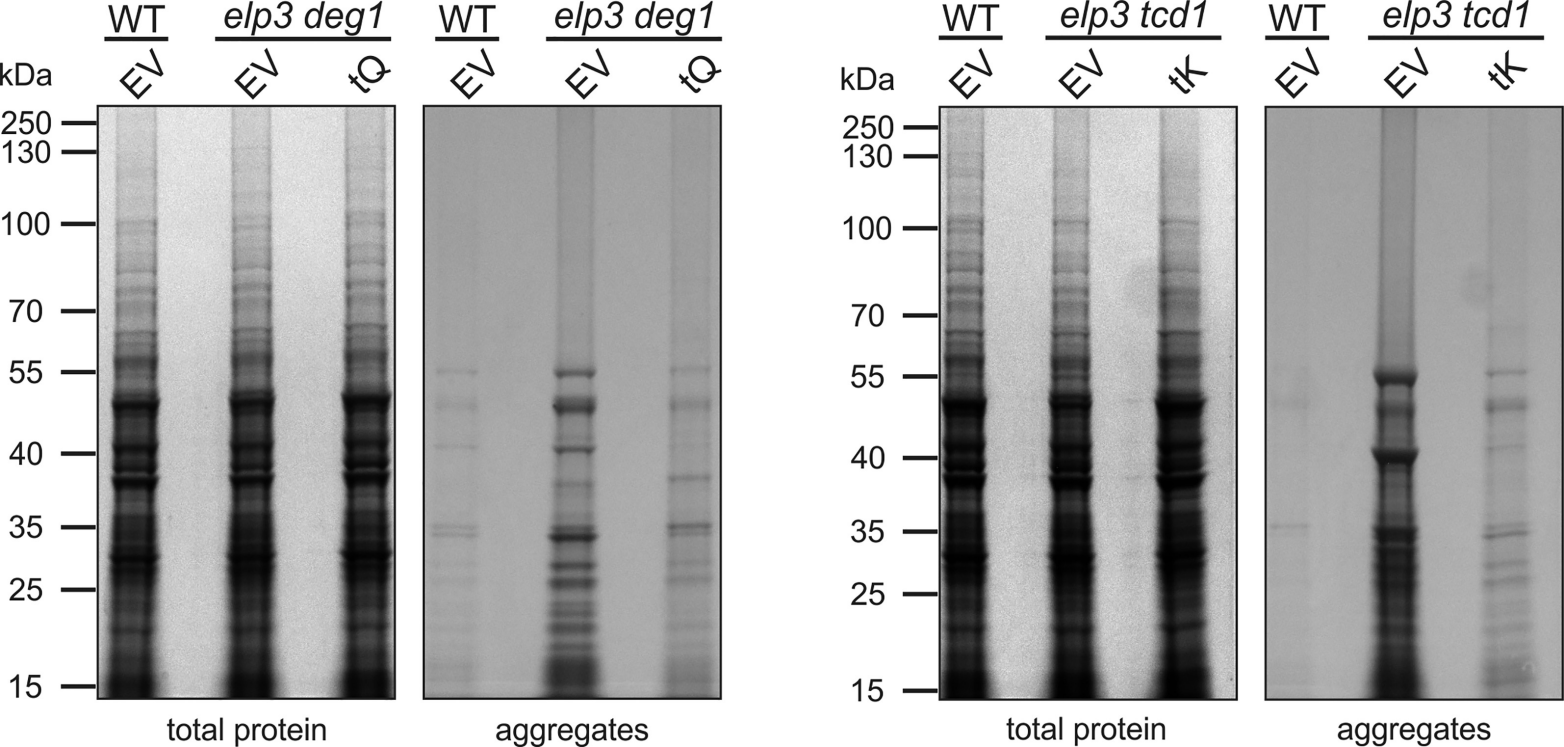
tRNA overexpression suppresses protein aggregation typical of different tRNA modification mutants. The indicated strains grown in minimal medium (YNB) and carrying empty vector (EV) or plasmids overexpressing tRNA^Gln^_UUG_ (tQ) or tRNA^Lys^_UUU_ (tK) were harvested in exponential growth phase and subjected to protein aggregation analysis as described above (see Figure 1).

Many phenotypes of tRNA modification mutants can be suppressed in yeast by overexpression of individual tRNAs (19,23,73,74). For instance, double mutants *urm1 deg1* and *elp3 deg1* are phenotypically rescued by overexpression of tRNA^Gln^_UUG_, while *tcd1 urm1* and *tcd1 elp3* mutants are suppressible by overexpression of tRNA^Lys^_UUU_ (20,47). We have further shown for these mutants that the tRNA which rescues growth defects also rescues protein biosynthesis of glutamine- or lysine-rich proteins (Supplementary Figure S1) (20). To test whether protein aggregation and transcriptomic changes observed in this study are a direct consequence of such translational defects, we examined whether both syndromes can be suppressed by tRNA overexpression. We isolated protein aggregates from *elp3 deg1* and *elp3 tcd1* cells carrying tRNA^Gln^_UUG_ or tRNA^Lys^_UUU_ overexpressing plasmids or empty vector controls, respectively. As expected (Figure 1F) and in line with an earlier report (20), both double mutants carrying empty vector displayed increased amounts of aggregated proteins (Figure 4). However, when the mutants expressed higher-than-normal levels of tRNA^Lys^_UUU_ (in *elp3 tcd1*) or tRNA^Gln^_UUG_ (in *elp3 deg1*), their propensity to form protein aggregates significantly dropped in relation to the empty vector controls (Figure 4).

**Figure 5.**
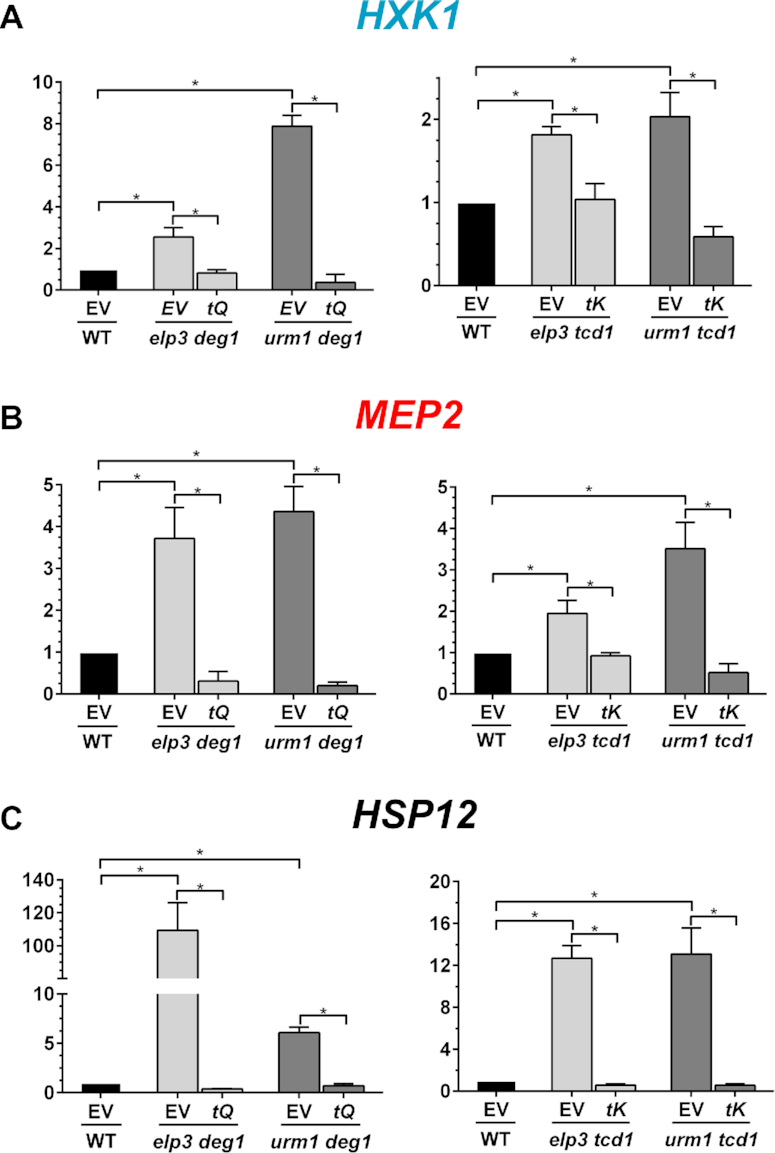
Higher-than-normal tRNA^Gln^_UUG_ or tRNA^Lys^_UUU_ doses suppress premature transcription of starvation/stationary marker genes. The indicated strains carrying empty vector (EV) or plasmids overexpressing tRNA^Gln^_UUG_ (tQ) or tRNA^Lys^_UUU_ (tK) were cultivated in YNB to the exponential growth phase and subjected to RNA isolation and qRT-PCR. Quantification of the relative mRNA levels of the marker genes *HXK1* (**A**), *MEP2* (**B**) and *HSP12* (**C**), respectively, was carried out as described above (Figure 2). Statistical significance was determined using the two-tailed *t*-test and indicated in the bar diagrams (* *P*≤ 0.05).

When checking for effects of tRNA overexpression onto premature starvation gene transcription seen above (Figure 2), *MEP2*, *HXK1* and *HSP12* quantification reconfirmed increased mRNAs levels in the modification mutants with empty vector (Figure 5A–C). However, overexpression of tRNA^Lys^_UUU_ or tRNA^Gln^_UUG_ significantly countered the induction of the three marker genes (Figure 5A–C), an observation showing that elevated tRNA levels not only rescue from increased protein aggregation but also suppress premature activation of starvation genes. Remarkably, the same tRNA suppressors (i.e. tRNA^Lys^_UUU_ or tRNA^Gln^_UUG_) were previously shown to rescue tRNA modification mutants from various stress induced growth phenotypes and restore translation efficiency or fidelity (14–16,19,20). Thus, induction of protein aggregation and premature starvation response activation are likely consequences of a defect in mRNA translation and proteostasis caused by loss of different anticodon modifications in distinct tRNA species.

### Loss of the ribosome associated chaperone complex induces expression of *HSP12* and *MEP2*

**Figure 6.**
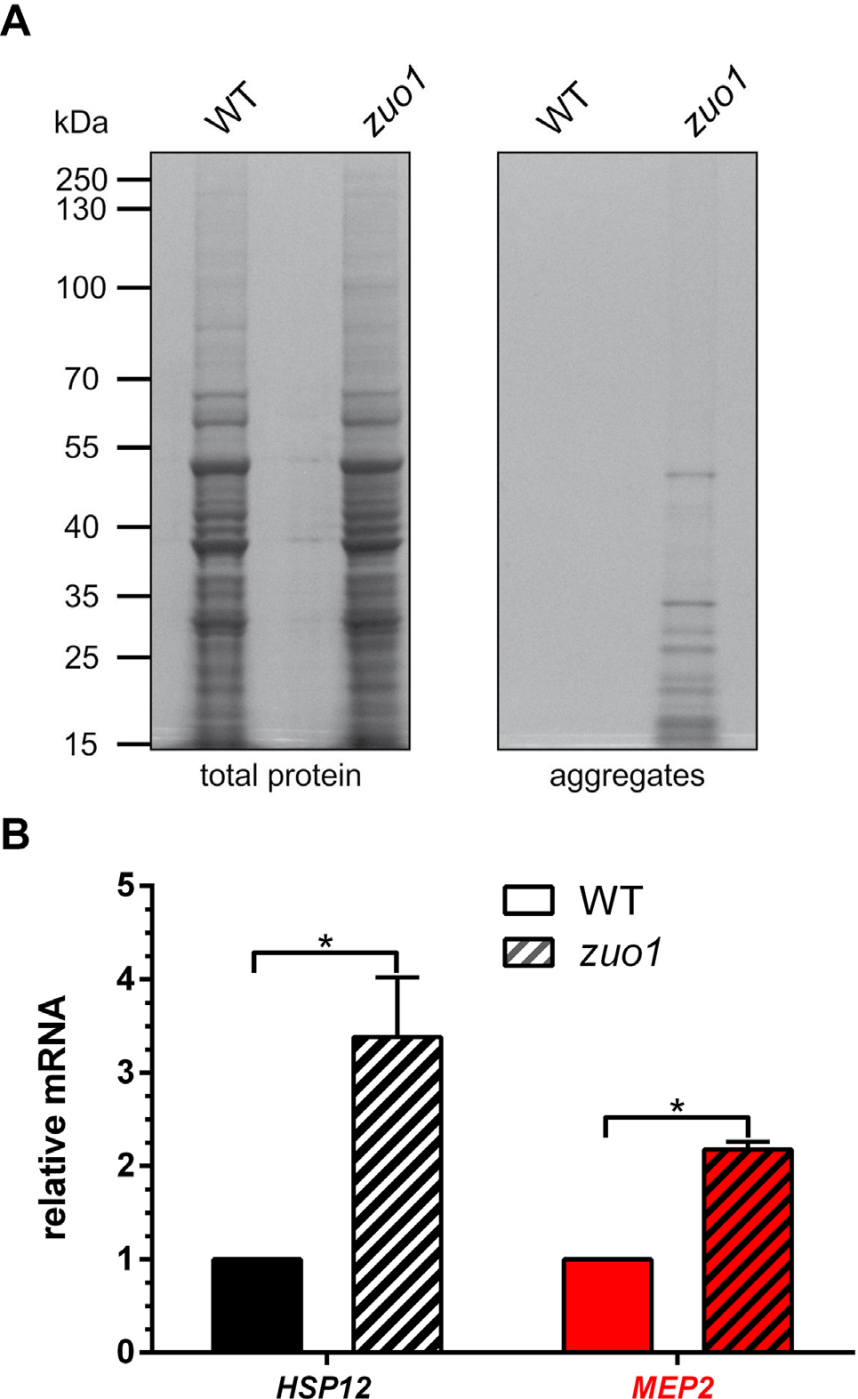
The deletion of *ZUO1* causes protein aggregation and induction of *HSP12* and *MEP2*. The indicated strains were cultivated in YPD-media until OD_600_ = 1.0, harvested and subjected to protein aggregate isolation (**A**) or total RNA isolation as described above (see Figures 1 and 2, respectively). (**B**) *HSP12* and *MEP2* marker gene mRNA levels were quantified, and the statistical significance was determined with two-tailed *t*-test indicated with asterisks (*P≤ 0.05).

To test whether protein aggregation itself can trigger expression of starvation genes, we utilized a strain deleted for *ZUO1* (zuotin) which encodes a Hsp40-like chaperone belonging to the ribosome-associated complex (RAC) (75,76). Together with the other RAC component Ssz1, zuotin supports folding of nascent peptide chains during translation and stimulates the activity of the Ssb1/Ssb2 chaperone (76). Hence, loss of *ZUO1* is expected to trigger protein aggregation in a tRNA modification independent manner and could be used to test the influence of protein aggregation on starvation marker gene expression. Indeed, we confirmed that *zuo1* mutants accumulate protein aggregates (Figure 6A). Next, we quantified mRNA levels of *HSP12* and *MEP2* in WT and the *zuo1* mutant. Notably, both genes are significantly induced in the *zuo1* mutant background (Figure 6B). We conclude that alterations in the propensity to form protein aggregates may be linked to the transcriptional aberrations observed in this study. Hence, protein aggregation may represent an unappreciated common trigger of starvation/stationary phase like transcriptional signatures caused by the absence of critical tRNA modifications.

## DISCUSSION

**Figure 7.**
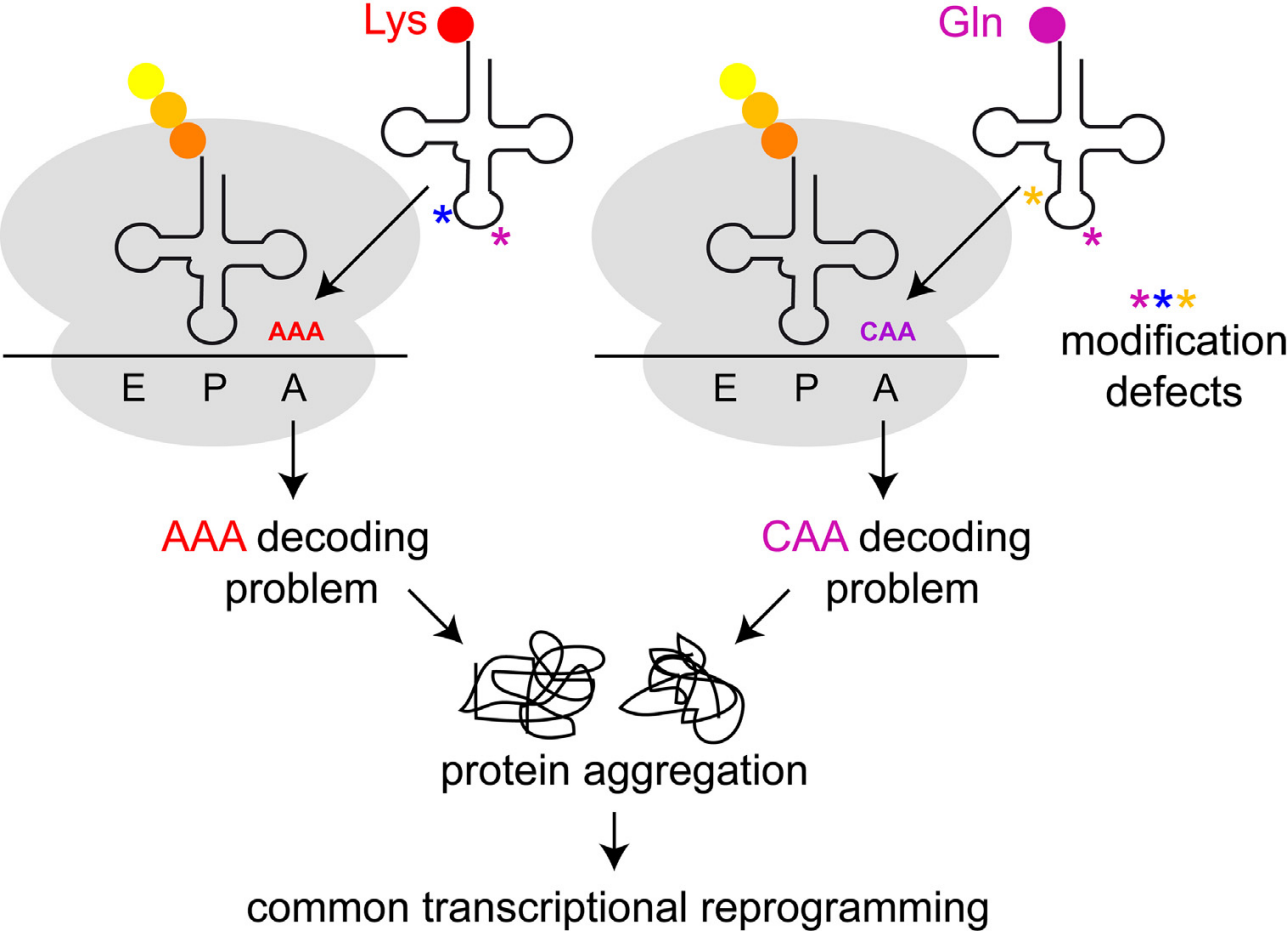
Model for the mechanistic links between specific decoding defects and protein aggregate induced transcriptional effects. Ribosomes with AAA (Lys) or CAA (Gln) codons in the A-site are depicted. These codons are difficult to decode when tRNA^Lys^UUU lacks mcm^5^/s^2^U and ct^6^A modifications (violet and blue asterisk) or when tRNA^Gln^UUG lacks mcm^5^/s^2^U and Ψ38 modifications (violet and yellow asterisk). Both conditions trigger the formation of protein aggregates which is assumed to mediate a common transcriptional reprogramming. Results obtained in this study suggest that protein aggregation per se rather than the depletion of specific proteins induces the common transcriptional effects associated with CAA or AAA decoding problems.

Negative interactions between different tRNA modification genes may be functionally linked to defects in few or even single tRNA species (20,74). In cases where modifications outside the ASL are lost, tRNA destabilization may account for the negative phenotype (74). However, when distinct ASL modifications are lost, tRNA remains stable but functionally impaired (2,15,77). This is well characterized for combined loss of mcm^5^U34 and s^2^U34, resulting in translational slowdown of the ribosome at CAA and AAA codons read by tRNA^Gln^_UUG_ and tRNA^Lys^_UUU_, respectively. One major consequence is an accumulation of protein aggregates, which are thought to cause the negative effects of the modification loss on cellular growth and stress resistance (15). Similarly, combined loss of mcm^5^/s^2^U34 and ct^6^A37 or Ψ38 modifications, which more specifically affect the functions of either tRNA^Lys^_UUU_ or tRNA^Gln^_UUG_, (20), enhances protein aggregation, presumably as a result of ribosomal slowdown at their cognate codons (20,78). Specific decoding defects of mRNA enriched in either AAA (Lys) or CAA (Gln) codons were demonstrated for the combined modification mutants and are consistent with the concept of modification tuneable transcripts (MoTTs) (Supplementary Figure S1) (20,79). When aggregate induction was compared between strains defective in mcm^5^s^2^U, mcm^5^U/ct^6^A and mcm^5^U/Ψ_38/39_, similar patterns were observed (Supplementary Figure S7). However, the latter strain, which is more severely affected in growth as compared to the other two (20), displayed a strong increase in protein aggregation (Supplementary Figure S7). Thus, protein aggregation may indeed play a key role in growth inhibition in the distinct tRNA modification mutants. Since mutants with composite tRNA modification defects affecting different tRNA species share striking similarities in cellular and morphological phenotypes (20), these are likely to be caused by a common mechanism. For example, if the formation of protein aggregates triggers a common phenotype, it may be irrelevant whether the aggregates are caused by decoding defects at CAA or AAA codons (Figure 7). However, if the phenotype is mediated by translational inefficiency of certain mRNAs (MoTTs), the outcome should be different in situations where distinct tRNAs are impaired because of loss or lack of anticodon modifications.

To gain further insight into this issue, we asked whether composite tRNA modification mutants selectively affecting tRNA^Gln^_UUG_ (i.e. *deg1* combinations) or tRNA^Lys^_UUU_ (i.e. *tcd1* combinations) share altered transcriptional responses. Indeed, the modification mutants with defects in tRNA^Gln^_UUG_ (i.e. *deg1 elp3, deg1 urm1*) and tRNA^Lys^_UUU_ (i.e. *tcd1 elp3, tcd1 urm1*) function, display overlapping aberrant transcriptional responses. Their commonality is an inappropriate transcriptional induction of multiple starvation pathways during exponential growth phase (Figures 1D, E, 2 and 5A–C). Transcriptome rearrangements resemble gene activation patterns normally executed upon nutrient depletion and entry into stationary phase (Figures 1 and 2) (50,51,59). Transcriptomes were profiled in cells grown to early exponential phase, a condition normally suppressing GAAC and NCR controlled genes and maintaining glucose repression as well as repression of common stationary phase genes such as *HSP12* (50,58). Markedly, we find that the different modification mutants significantly derepress genes from these different categories and additionally induce respiratory genes (Figure 1) under nutrient replete conditions. Since these are defects in common with all mutant backgrounds examined, they are triggered by decoding defects in distinct tRNAs. In addition, we also observed premature activation of autophagy, a starvation response usually requiring inhibition of TORC1 signalling. This is in further support of our conclusion that in yeast, broad starvation responses typical of stationary phase can be inappropriately triggered by loss of different anticodon loop modifications in distinct tRNA species.

Derepression of GAAC controlled genes has been observed in different tRNA modification mutants including mcm^5^U34 and s^2^U34 deficient strains (13,15,27,49,66,80). Canonical GAAC pathway induction occurs via enhanced Gcn4 translation upon Gcn2 activation by uncharged tRNA (81). Absence of the mcm^5^U34 and s^2^U34 modifications did not affect steady state levels of charged tRNA and has been shown to induce GAAC controlled genes in a Gcn2 independent manner (13,26). Hence, a non-canonical GAAC induction was proposed for the U34 modification mutants (13). In further support, the set of double mutants examined in this study displayed a rather ambivalent than uniform modulation of GAAC controlled genes, including a strong downregulation of individual Gcn4 dependent amino acid biosynthesis genes. This argues against a canonical induction of Gcn4 dependent amino acid biosynthesis. For example, in the *tcd1 elp3* mutant, *LYS2, LYS9* and *LYS20* genes of the lysine biosynthesis pathway are upregulated, whereas *LYS1* and *LYS14* are downregulated (Supplementary Figure S4) and all of these are regulated by Gcn2/Gcn4 (82). Similar deregulations in opposing directions were observed in the other mutants and in additional amino acid biosynthesis pathways. Interestingly, mcm^5^U34 deficiency was shown to result in elevated levels of lysine but reduced levels of several other amino acids (25). Hence, the GAAC deregulation observed in this study may be consistent with a general loss of regulatory control over amino acid biosynthesis in different tRNA modification mutants. It should be noted that other conditions for tRNA charging independent Gcn4 upregulation have been described. For example, loss of the tRNA nuclear exporter Los1 results in reduced cytoplasmic availability of tRNA and triggers upregulation of Gcn4 dependent amino acid biosynthesis genes (83). In addition, ribosomal slow down due to depletion of a specific tRNA in mice has been shown to trigger GAAC activation (84). Hence, effects of various tRNA modification defects on the regulatory control over Gcn4 dependent gene expression may similarly involve reduced availability of fully functional tRNA for translation.

In addition to GAAC deregulation, we observed strong indications for TORC1 inhibition and loss of repression of stationary phase genes, including *HSP12* and *HXK1*. *HSP12* expression has been found to partly depend on the activity of TORC1 (85–87). To further investigate whether upregulation of glucose repressed genes could be linked to TORC1 inhibition, we examined *HXK1* and *MEP2* expression upon either deletion of *TOR1* or application of L-methionine sulfoximine (MSX). The latter depletes glutamine which is monitored by TORC1 (68,88). We find that both *TOR1* deletion and MSX treatment specifically upregulate expression of the NCR gene *MEP2* without affecting *HXK1* mRNA levels (Supplementary Figure S6). Hence, *HXK1* upregulation by different tRNA modification defects (and, by extension, loss of glucose repression in general) may occur independent of TORC1 inhibition. In this context, it is noteworthy that the transcriptional repressor genes *CYC8, NRG1* and *HXK2* are commonly downregulated in each of our combined tRNA modification mutants. Since the gene products are involved in repression of various genes including *HXK1* and other glucose repressed genes (52–54), their downregulation may be relevant for loss of glucose repression in the modification mutants.

In s^2^U34 deficient strains, increased storage carbohydrate synthesis resulting in accumulation of trehalose and glycogen was in fact detected, representing another inadequate starvation response that is normally suppressed in the presence of glucose. However, the mechanism appeared to depend on metabolic deregulation rather than transcriptional changes (89). Since our combined tRNA modification mutants display much stronger growth phenotypes than s^2^U34 deficient mutants (20), we anticipate even further enhanced effects on their metabolic states. The strong upregulation of trehalose and glycogen biosynthesis genes *TSL1*, *GSY2* and *GLC3* in each of the double mutants further supports this interpretation.

Since protein aggregation is a common consequence in different tRNA modification mutants with distinct tRNA substrates and is suppressible by tRNA overexpression (Figure 4), protein aggregates might be relevant for the induction of transcriptional starvation responses. In support, we demonstrated that induced expression of starvation markers in the different modification mutants is suppressed by overexpression of the same tRNAs that suppress protein aggregation (Figure 5). In addition, the protein aggregation prone mutant *zuo1* induces *HSP12* and *MEP2* gene activation which might suggest a transcriptional alteration comparable to the tRNA modification mutants (Figure 6). Therefore, protein aggregation might represent a trigger for the altered transcriptional programs observed in the tRNA modification mutants. In addition, the proteasome capacity is known to influence expression of *HXK1* and elevated proteasome activity impacts on the abundance of the Mig1 repressor, resulting in prematurely activated respiration and loss of glucose repression (90). If protein aggregates accumulating in combined tRNA modification mutants are handled by the proteasome, its capacity for turn-over of transcription factors may be lowered. Hence, in addition to the transcriptional repression of *CYC8, NRG1* and *HXK2* observed in the different tRNA modification mutants, altered turnover-rates of transcription factors may be involved in the common starvation responses.

Interestingly, in addition to cytosolic chaperones and the proteasome also mitochondria appear to play a crucial role in the maintenance of proteostasis (91,92). Cytosolic aggregating proteins were shown to stimulate mitochondrial biogenesis and are imported into mitochondria for subsequent degradation. It was suggested that aggregate overload may damage mitochondrial function and that reduced mitochondrial activity impairs cytosolic proteostasis (91). Hence, the induced expression of genes related to mitochondria function in the aggregation prone tRNA modification mutants and impaired respiratory growth could be linked to mitochondrial aggregate overload.

Different possibly overlapping mechanisms might account for the induction of protein aggregation in the distinct modification mutants. On the one hand, decoding defects induced by loss of critical modifications might alter the acceptance of incorrect tRNAs by the ribosome or affect reading frame maintenance, resulting in formation of aberrant polypeptides. Indeed, influences of U34 and A37 modification defects on codon misreading and ribosomal frameshift were observed using reporter gene based assays (14,16,17). Hence, an increased rate of translational error could account for elevated protein instability. In fact, low level mistranslation in yeast expressing a leucine decoding tRNA^Ser^ resulted in transcriptional induction of a proteotoxic stress response including heat shock proteins such as Hsp12 and Hsp26, which are also induced in our combined tRNA modification mutants (93). Interestingly, the transcriptional response to induced mistranslation exhibits further similarities with the changes observed in the tRNA modification mutants. In both cases, carbohydrate metabolic genes subject to glucose repression (such as *TSL1* and *ALD3*) are commonly induced, further supporting the idea, that part of the transcriptional changes observed in our tRNA modification mutants might be attributable to changes in translational fidelity. On the other hand, decoding defects may affect the co-translational folding of the nascent polypeptide and result in elevated protein aggregation. We have provided evidence that the different combined mutants analysed in this study exhibit a decoding defect of either tRNA^Lys^_UUU_ (Supplementary Figure S1) or tRNA^Gln^_UUG_ (20), resulting in protein aggregation (Figure 4), which likely mediates the transcriptional responses (Figure 7).

In summary, our study demonstrates that cellular responses to loss of tRNA modifications are more complex than previously anticipated and likely include mechanisms beyond post-transcriptional regulation. These may include detection of hypomodified tRNA by nutrient sensing and signalling pathways (such as TORC1 and GAAC) (68,89). However, in our view, a major contribution likely originates from defects in mRNA translation elongation that compromise protein homeostasis. Future work will be required to delineate whether aggregate induced transcriptome alterations also contribute to the various neuropathies associated with tRNA modification defects in humans.

## DATA AVAILABILITY

Sequencing data are available at the Gene Expression Omnibus (accession number GSE141887).

## Supplementary Material

gkaa455_Supplemental_Files

## Notes

Present address: Teresa Laguna, Computational Biology group, IMDEA Food Institute, Ctra. Cantoblanco 8, 28049 Madrid, Spain.
