# Supplementary material for: Misactivation of multiple starvation responses in yeast by loss of tRNA modifications
Source: Nucleic Acids Res. 2020 Jun 2;48(13):7307–20. doi: 10.1093/nar/gkaa455 (PMC7367188; doi:10.1093/nar/gkaa455)
Supplement: gkaa455_Supplemental_Files [file gkaa455_supplemental_files.zip › Bruch_et_al_SupMat_revised2.docx]

**Supplemental Information**

**Misactivation of multiple starvation responses in yeast by loss of tRNA modifications**

Alexander Bruch^1^, Teresa Laguna^2,3^, Falk Butter^2^, Raffael Schaffrath ^1,^* and Roland Klassen ^1,^*

^1^ Institut für Biologie, Fachgebiet Mikrobiologie, Universität Kassel,

Heinrich-Plett-Str. 40, D-34132 Kassel, Germany

^2^ Department of Quantitative Proteomics, Institute of Molecular Biology,

Ackermannweg 4, D-55128 Mainz, Germany

^3^ Present address: Computational Biology group, IMDEA Food Institute,

Ctra. Cantoblanco 8, 28049 Madrid, Spain

* Co-corresponding authors:

Raffael Schaffrath ([schaffrath@uni-kassel.de](javascript:linkTo_UnCryptMailto('wksvdy4cmrkppbkdrJexs7ukccov8no');))

Lead contact: Roland Klassen (roland.klassen@uni-kassel.de)

Phone: +49-561-804-4340 FAX: +49-561-804-4337

Keywords:

tRNA modifications, TOR pathway, autophagy, starvation responses

**Supplemental Tables**

Table S1. *S. cerevisiae* strains used or generated throughout this study.

| **Strain** | **Genotype** | **Reference/source** |
| --- | --- | --- |
| BY4741 | *MAT***a**, *his3Δ, leu2Δ, met15Δ, ura3Δ* | Euroscarf, Frankfurt |
| RK520 | BY4741 *ncs2Δ::SpHIS5 elp6Δ::KanMX4* | (Bruch et al., 2018) |
| JF127 | BY4741 *elp3Δ:: loxP tcd1Δ:: KanMX4* | this study |
| JF129 | BY4741 *urm1Δ:: loxP tcd1Δ:: KanMX4* | this study |
| JF130 | BY4741 *urm1Δ::KanMX4 deg1Δ::loxP* | this study |
| RK220 | BY4741 *elp3Δ::KanMX4 deg1Δ::SpHIS5* | (Klassen et al., 2016) |
| AB102 | BY4741 *urm1Δ::KanMX4 deg1Δ::loxP atg1Δ::KlURA3* | this study |
| Y06864 | BY4741 *tor1Δ::KanMX4* | Euroscarf, Frankfurt |
| AB10 | BY4741; *SRP21-(HA)6::HIS3MX6* | this study |
| AB11 | BY4741; *urm1Δ::loxP; tcd1Δ::kanMX4; SRP21-(HA)6::HIS3MX6* | this study |
| AB12 | BY4741; *elp3Δ::loxP; tcd1Δ::kanMX4; SRP21-(HA)6::HIS3MX6* | this study |
| AB14 | BY4741; *tcd1Δ::kanMX4; SRP21-(HA)6::HIS3MX6* | this study |

Table S2. Oligonucleotides used in this study.

| **Oligonucleotide** | **Sequence (5´ - 3´)** | **Target** | **Reference/source** |
| --- | --- | --- | --- |
| ATG1_KO_Fwd | ACCCCATATTTTCAAATCTCTTTTACAACACCAGACGAGAAATTAAGAAACAGCTGAAGCTTCGTACGC | pUG72  /*ATG1* | this study |
| ATG1_KO_Rev | AAATATAGCAGGTCATTTGTACTTAATAAGAAAACCATATTATGCATCACGCATAGGCCACTAGTGGATCTG | pUG72  /*ATG1* | this study |
| ATG1_KO+_Fwd | GGCAAAGGAGATAGGAGAATA | *ATG1* | this study |
| ATG1_KO+_Rev | CGTAAAGCATTTCGAGAGTA | *ATG1* | this study |
| pUG72/KlURA fw | TATCACCAACGCCCACGGG | *KlURA3* | this study |
| HXK1_qPCR_Fwd | GCCTCATACTACACTGACCCAG | *HXK1* | this study |
| HXK1_qPCR_Rev | TCGTCTGCTAATTTGCCCTCC | *HXK1* | this study |
| HSP12_qPCR_Fwd | CATCACTGACAAGGCCGACA | *HSP12* | this study |
| HSP12_qPCR_Rev | CGTTCAACTTGGACTTGGCG | *HSP12* | this study |
| *MEP2*-qFW | GTATGTTTGCCGCAGTCACC | *MEP2* | (Scheidt et al., 2014) |
| *MEP2*-qRV | CAGACCCAGCATGCAATAGG | *MEP2* | (Scheidt et al., 2014) |
| qPCR_ACT1_FW | TTCCAGCCTTCTACGTTTCC | *ACT1* | (Klassen et al., 2016) |
| qPCR_ACT1_RV | AATCTCTACCGGCCAAATCG | *ACT1* | (Klassen et al., 2016) |
| S3_Srp21_Fwd | TCTGCAGCTTCAAAAAAGAAAAAAAATAAAAACAAGGGCAAAAAAAAGCGTCGTACGCTGCAGGTCGAC | pYM15/*SRP21* | this study |
| S2_Srp21_Rev | TATATAGTATTTTATTTCATATTCAATCTCCTAGTATACATGAAATATTTAATCGATGAATTCGAGCTCG | pYM15/*SRP21* | this study |
| Srp21_Tag+_Fwd_II | TGTGCTGTCTTCGATTGACC | *SRP21* | this study |
| Srp21_Tag+_Rev | TCTAGGATAACGGCGCAAAC | *SRP21* | this study |

Table S12. Overall induction of glucose-supressed genes necessary for gluconeogenesis and trehalose biogenesis. Only significant log2-fold changes are displayed (adj. *p* ≤ 0.01).

| **Genes** | ***elp3 deg1*** | ***urm1 deg1*** | ***elp3 tcd1*** | ***urm1 tcd1*** |
| --- | --- | --- | --- | --- |
| *HXK2* | -0,43 | -1,57 | -1,59 | -1,02 |
| *TSL1* | 0,64 | 0,84 | 0,92 | 0,70 |
| *GSY2* | 0,79 | 1,33 | 1,26 | 0,79 |
| *GLC3* | 1,01 | 2,24 | 1,82 | 1,07 |
| *TPS1* | 1,01 | 0,87 | 0,99 | - |
| *TPS2* | - | 0,51 | 0,72 | - |
| *MAE1* | 0,13 | - | 0,80 | 1,40 |
| *PYC1* | -0,56 | -1,48 | -0,32 | -0,26 |
| *PYC2* | -0,27 | -1,31 | - | - |
| *MDH2* | -1,33 | -0,73 | - | - |
| *PCK1* | -0,68 | -0,74 | - | - |
| *ENO1* | 1,97 | 0,75 | - | 0,89 |
| *ENO2* | 1,15 | -0,12 | - | 0,87 |
| *GPM1* | 1,67 | 0,79 | 0,94 | 1,73 |
| *PGK1* | 1,65 | 0,12 | - | 0,81 |
| *TDH1* | 1,06 | 0,46 | - | 0,98 |
| *TDH2* | 1,61 | 0,63 | - | 0,82 |
| *TDH3* | 1,39 | -0,32 | 0,50 | 0,60 |
| *FBA1* | 1,39 | 0,43 | 1,16 | 1,93 |
| *FBP1* | -1,12 | - | - | - |
| *PGI1* | 0,92 | - | - | 0,52 |

Table S13. Log2-fold changes of genes assigned to the GO-term “ATP metabolism” (adj. *p* ≤ 0.01) relevant during respiration of the indicated four double mutants.

| **Genes** | ***elp3 deg1*** | ***urm1 deg1*** | ***elp3 tcd1*** | ***urm1 tcd1*** |
| --- | --- | --- | --- | --- |
| *QCR9* | 0,78 | 0,85 | 0,97 | 0,70 |
| *COX6* | 0,72 | 0,45 | 0,54 | 0,52 |
| *COX4* | 1,61 | 1,35 | 1,26 | 1,27 |
| *COX5B* | 1,42 | 0,56 | 1,00 | 0,62 |
| *ATP7* | 0,80 | 0,32 | 0,79 | 1,33 |
| *ATP3* | 0,97 | 0,66 | 0,32 | 0,50 |
| *ATP14* | 0,82 | 0,30 | 0,42 | 0,80 |
| *GPM1* | 1,67 | 0,79 | 0,94 | 1,73 |
| *RIP1* | 0,98 | 0,60 | 0,51 | 0,71 |
| *SDH1* | 0,77 | 0,34 | 0,66 | 1,11 |
| *COX8* | 0,96 | 0,64 | 0,82 | 0,75 |
| *GLK1* | 1,47 | 0,87 | 1,08 | 0,70 |
| *COX12* | 1,31 | 0,85 | 1,32 | 1,28 |
| *COX7* | 1,27 | 0,61 | 1,19 | 1,31 |
| *ATP19* | 1,60 | 1,09 | 1,49 | 1,60 |
| *ERR3* | 1,39 | 1,14 | 1,73 | 1,28 |
| *ATP4* | 0,80 | 0,63 | 0,63 | 0,83 |
| *QCR10* | 1,25 | 0,73 | 1,13 | 1,29 |
| *CYT1* | 1,60 | 1,23 | 1,09 | 1,18 |
| *ATP17* | 0,96 | 0,40 | 0,81 | 1,14 |
| *COX13* | 1,05 | 0,82 | 1,17 | 1,27 |
| *ATP16* | 1,02 | 0,59 | 0,52 | 0,81 |
| *COX5A* | 0,46 | 0,57 | 0,71 | 0,81 |
| *TIM11* | 1,15 | 0,98 | 1,23 | 1,26 |
| *FBA1* | 1,39 | 0,43 | 1,16 | 1,93 |

Table S14. TORC1 controlled genes involved among others in NCR are indicated with significant log2-fold changes (adj. *p*≤ 0.01).

| **Genes** | ***elp3 deg1*** | ***urm1 deg1*** | ***elp3 tcd1*** | ***urm1 tcd1*** |
| --- | --- | --- | --- | --- |
| *DAL3* | 2,20 | 1,71 | 1,86 | 1,97 |
| *GAT1* | 1,61 | 1,72 | 1,47 | 1,91 |
| *GDH1* | 2,17 | 1,17 | 1,02 | 1,08 |
| *GLN1* | 0,80 | 0,25 | 0,59 | 0,98 |
| *MEP2* | 2,15 | 2,58 | 2,75 | 3,47 |
| *MEP3* | 0,51 | 0,19 | 0,86 | 1,11 |
| *DAL80* | 1,61 | 2,55 | - | 3,51 |
| *GLN3* | -0,62 | -0,86 | -0,39 | -0,41 |
| *MTC5* | 0,72 | 0,67 | 0,53 | 0,48 |
| *RPS6B* | 0,96 | 0,76 | 0,46 | 0,79 |
| *WHI2* | 0,20 | 0,53 | 0,54 | 0,49 |

Table S15. Expression pattern of the enlisted genes encoding chaperones and heat shock proteins according to the GO-term “protein folding” (adj. *p* ≤ 0.01) in the indicated tRNA modification mutants. Alteration of the transcript levels are indicated in log2-fold changes for every strain.

| **Genes** | ***elp3 deg1*** | ***urm1 deg1*** | ***elp3 tcd1*** | ***urm1 tcd1*** |
| --- | --- | --- | --- | --- |
| *SSA2* | -0,96 | -1,43 | -1,38 | -0,99 |
| *SSE1* | -0,85 | -0,92 | -0,71 | -0,50 |
| *SSA1* | -0,69 | -1,25 | -0,40 | -0,44 |
| *TCM62* | -0,85 | -0,72 | -0,77 | -0,58 |
| *MDJ1* | -1,14 | -1,09 | -0,72 | -0,54 |
| *HSP78* | -0,66 | -1,29 | -0,41 | -0,41 |
| *SSC1* | -0,21 | -0,21 | -0,24 | -0,21 |
| *ZUO1* | -0,31 | -0,20 | -0,62 | -0,48 |
| *STI1* | -1,06 | -1,43 | -0,97 | -0,85 |
| *HSP82* | -1,70 | -1,77 | -0,97 | -0,75 |
| *SIS1* | -1,08 | -1,33 | -1,04 | -0,72 |
| *SSA4* | -3,45 | -1,90 | -1,37 | -1,14 |
| *HSP42* | -1,84 | -1,79 | -0,55 | -0,58 |
| *CCT8* | -0,22 | -0,24 | -0,39 | -0,25 |
| *SSZ1* | -0,39 | -0,48 | -0,44 | -0,39 |
| *HSP104* | -1,25 | -1,32 | -0,51 | -0,70 |
| *BTN2* | -3,18 | -2,90 | -1,45 | -0,86 |
| *CIN1* | -1,33 | -1,18 | -0,92 | -0,76 |
| *PAC10* | -1,05 | -0,85 | -0,94 | -1,17 |
| *HSC82* | -0,58 | -0,70 | -1,05 | -1,00 |
| *YDJ1* | -0,42 | -0,67 | -0,43 | -0,32 |
| *CUR1* | -0,64 | -1,10 | -0,70 | -0,84 |
| *CPR6* | -1,09 | -1,38 | -1,18 | -1,11 |
| *APJ1* | -1,99 | -1,72 | -1,24 | -0,97 |
| *HSP60* | -0,36 | -0,63 | -0,43 | -0,36 |
| *HSP12* | 4,50 | 4,92 | 4,94 | - |
| *HSP26* | 0,81 | 1,64 | 2,79 | 2,34 |

**Supplemental Figures**


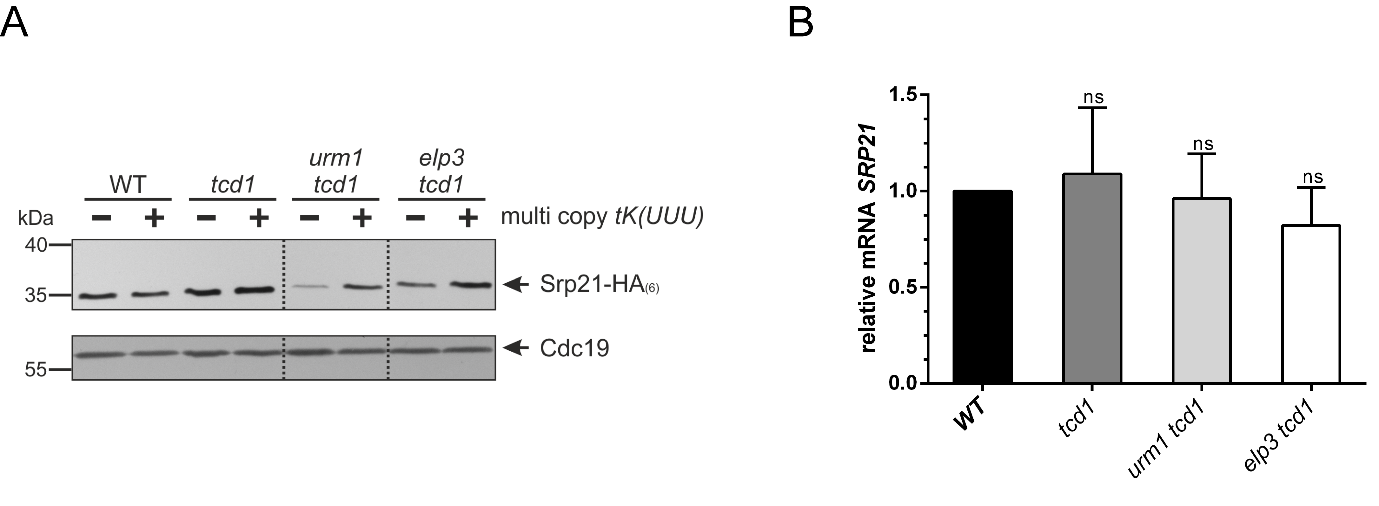


Figure S1. Defective translation of lysine-rich *SRP21* is rescued by tRNA^Lys^_UUU_ overexpression in *tcd1* mutant combinations. (A) The indicated strains expressing 6xHA-tagged Srp21 were transformed with either empty vector (-) or tRNA^Lys^_UUU_ (*tK(UUU)*) overexpressing plasmid (+) and cultivated in YNB medium until exponential growth phase. Detection of Srp21 and Cdc19 (loading control) was achieved by anti-HA or anti-Cdc19 antibodies, respectively. (B) Quantification of *SRP21* and *ACT1* mRNA levels in the indicated strains by qRT-PCR. Significance of differences in mRNA levels compared to the wild type was tested utilizing two tailed *t-*test (ns: not significant, *p*>0.05).


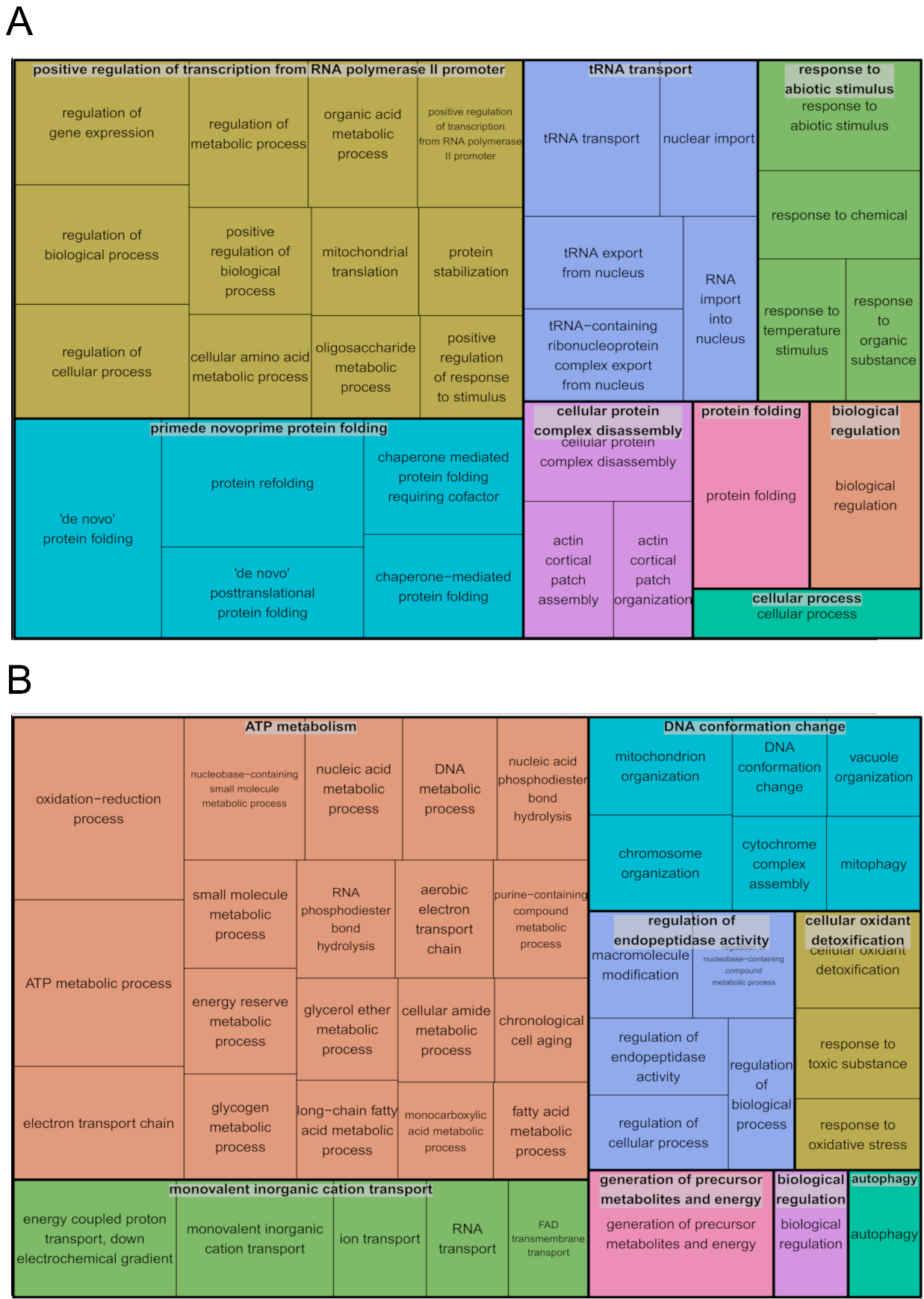


Figure S2. Gene ontology (GO) analysis for genes commonly deregulated in tRNA modification mutants (*elp3 deg1*, *urm1 deg1*, *elp3 tcd1* and *urm1 tcd1*). Overlapping downregulated (A) and upregulated genes (B) were assigned for their cellular function with GO slim mapper and term finder function (Harris et al., 2008) of SGD (*Saccharomyces* genome database) (Skrzypek and Hirschman, 2011). Results were processed with REVIGO (Supek et al., 2011) and displayed by treemaps.


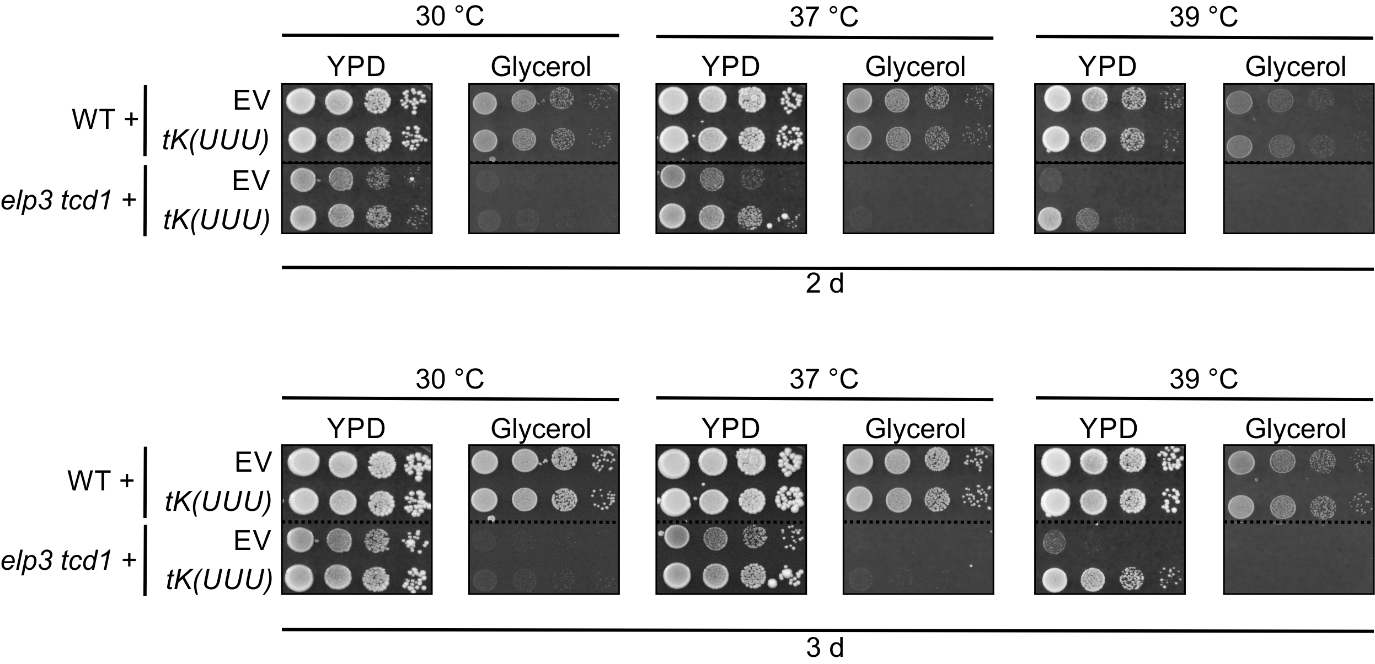


Figure S3. Respiratory growth defect of a tRNA modification double mutant and response to tRNA overexpression. Wild type (WT) and the *elp3 tcd1* mutant were each transformed with empty vector (EV) or the tRNA^Lys^_UUU_ (*tK(UUU)*) overexpression construct, serially diluted and replica spotted on YPGlycerol (2%) and YPD plates and incubated at 30°C, 37°C or 39°C for 2 or 3 days, respectively.


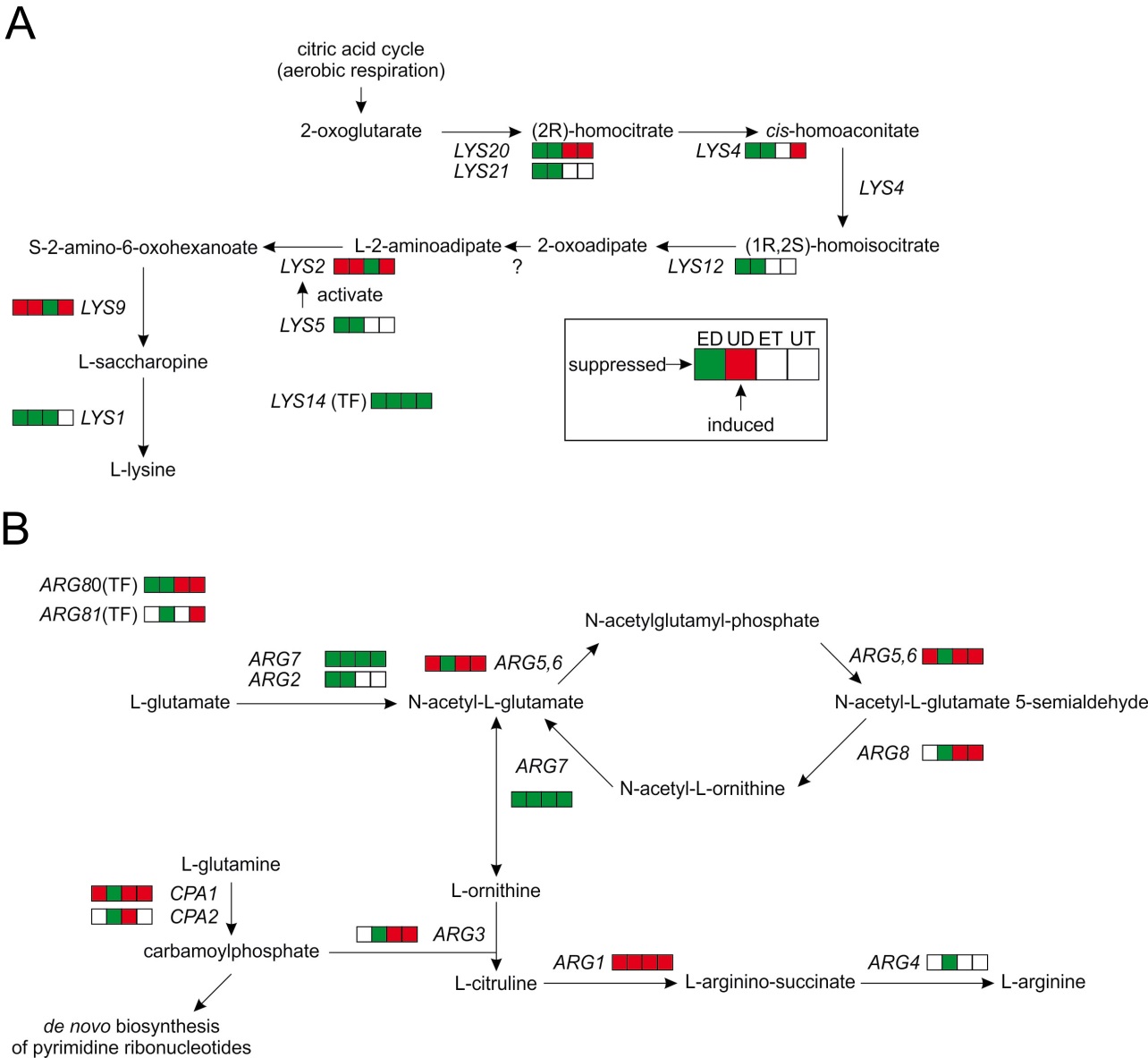


Figure S4. Genes involved in amino acid biosynthesis are commonly deregulated rather than collectively induced in tRNA modification mutants. All genes involved in lysine (A) or arginine (B) biosynthesis in yeast are indicated at every step of the pathways. The given boxes at every gene (order: *elp3 deg1*=ED, *urm1 deg1*=UD, *elp3 tcd1*=ET, *urm1 tcd1*=UT) display induction (red)/suppression (green) of the respective gene and tRNA modification mutant based on the transcriptome analysis while blank boxes represent no available data. TF: transcription factor/transcriptional activator. Taken and adapted from SGD (Skrzypek and Hirschman, 2011).


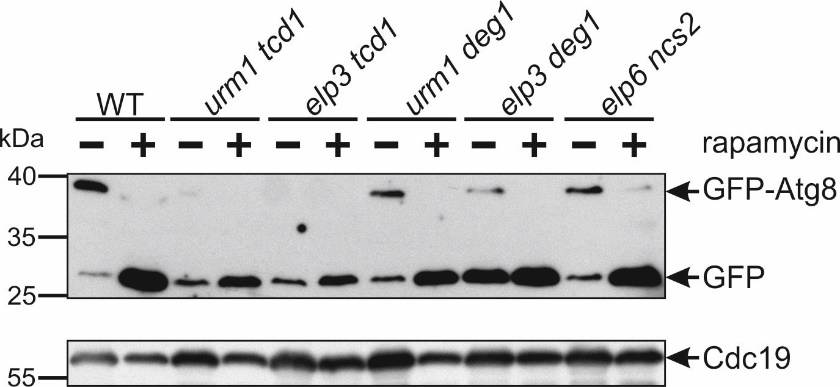


Figure S5. Effect of rapamycin treatment on autophagy induction in tRNA modification mutants compared to wild type (WT) cells. All indicated strains were cultivated until exponential growth phase (OD_600_=1.0) in the absence (-) or presence (+) of rapamycin (0.2 µg/mL). To examine GFP-Atg8 proteolysis, equal amounts of protein extracts were subjected to Western analysis using anti-GFP and anti-Cdc19 antibodies for detection of GFP or Cdc19 (see also Fig 3C).


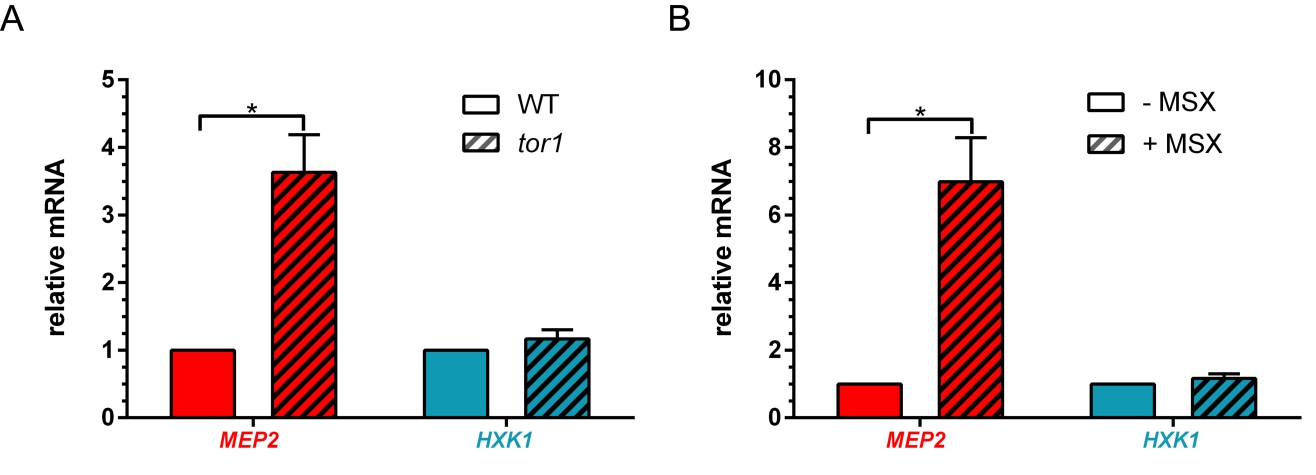


Figure S6. *TOR1* gene deletion or L-methionine sulfoximine (MSX) treatment leads to NCR pathway (*MEP2*) induction without affecting of target glucose reperession (*HXK1*). mRNA quantification of the different genes involved wild-type (WT) and *tor1* mutant (A) or 10 mM MSX treated (+) or untreated (-) WT-cells (B) cultivation, RNA isolation and qRT-PCR as described (see Fig. 2). Statistical significance (*p* ≤ 0.05) is indicated by the asterisks.


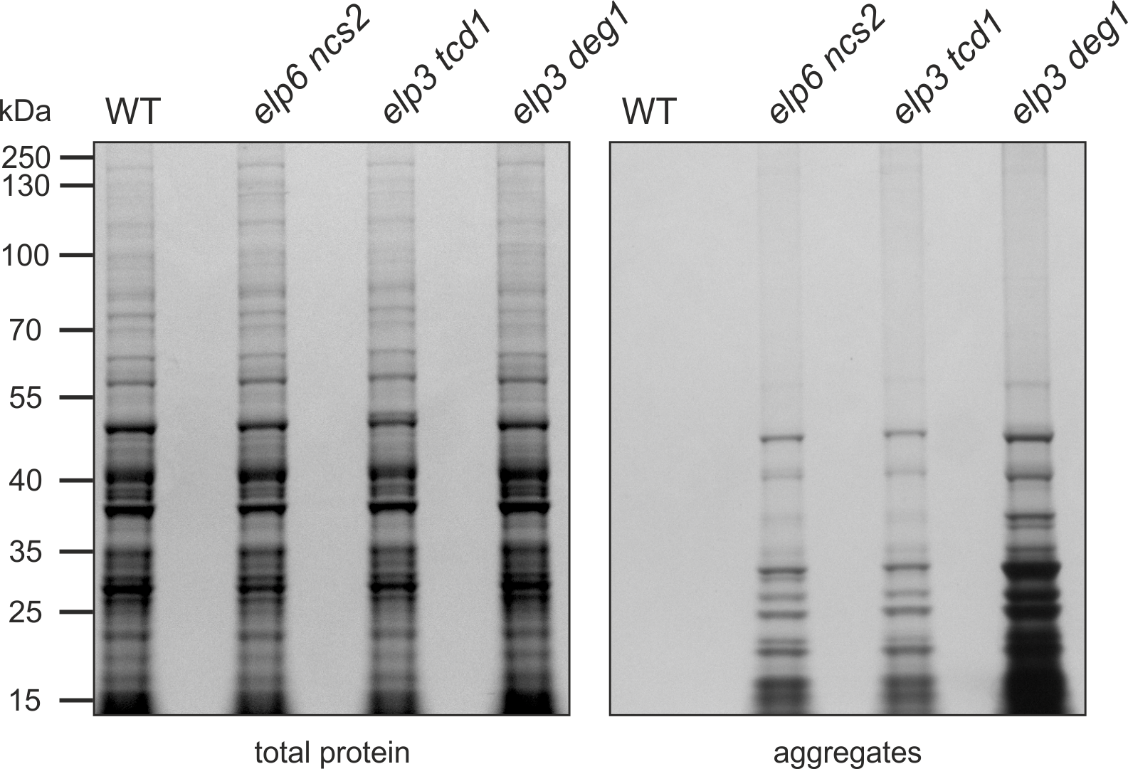


Figure S7. Comparison of protein aggregate formation in composite tRNA modification mutants *elp6 ncs2*, *elp3 tcd1* and *elp3 deg1*. The indicated strains were cultivated in YPD media until exponential growth phase (OD_600_=1.0), harvested and subjected to protein aggregate isolation detailed in Fig. 1.

**References:**

Klassen,R., Ciftci,A., Funk,J., Bruch,A., Butter,F. and Schaffrath,R. (2016) tRNA anticodon loop modifications ensure protein homeostasis and cell morphogenesis in yeast. *Nucleic Acids Res.*, **44**, 10946–10959.

Bruch,A., Klassen,R. and Schaffrath,R. (2018) Unfolded protein response suppression in yeast by loss of tRNA modifications. *Genes (Basel).*, **9**, 13–16.

Scheidt,V., Juedes,A., Baer,C., Klassen,R. and Schaffrath,R. (2014) Loss of wobble uridine modification in tRNA anticodons interferes with TOR pathway signaling. *Microb. Cell*, **1**, 416–424.

Harris,M.A., Deegan,J.I., Ireland,A., Lomax,J., Ashburner,M., Tweedie,S., Carbon,S., Lewis,S., Mungall,C., Day-Richter,J., *et al.* (2008) The Gene Ontology project in 2008. *Nucleic Acids Res.*, **36**, 440–444.

Supek,F., Bošnjak,M., Škunca,N. and Šmuc,T. (2011) REVIGO summarizes and visualizes long lists of gene ontology terms. *PLoS One*, **6**, e21800.

Skrzypek,M.S. and Hirschman,J. (2011) Using the Saccharomyces Genome Database (SGD) for analysis of genomic information. *Curr. Protoc. Bioinformatics*, **Chapter 1**, Unit 1.20.1-23.
